# An index of the initial blood pressure response to angiotensin II treatment and its association with clinical outcomes in vasodilatory shock

**DOI:** 10.1186/s13054-025-05311-z

**Published:** 2025-02-19

**Authors:** Daniel E. Leisman, Patrick M. Wieruszewski, Laurence W. Busse, Lakhmir S. Chawla, Kathryn A. Hibbert, Damian R. Handisides, Ashish K. Khanna, Marlies Ostermann, Michael T. McCurdy, Christopher D. Adams, Tony N. Hodges, Rinaldo Bellomo, the ATHOS-3 Investigators

**Affiliations:** 1 Division of Pulmonary and Critical Care Medicine, Massachusetts General Hospital, 55 Fruit St., Bulfinch 148, Boston, MA USA; 2 Division of Pulmonary, Critical Care, and Sleep Medicine, Beth Israel Deaconess Medical Center, Boston, MA 02114 USA; 3 Department of Pharmacy, Mayo Clinic, Rochester, MN USA; 4 Department of Anesthesiology, Mayo Clinic, Rochester, MN USA; 5 Department of Medicine, Emory University, Atlanta, GA USA; 6 Emory Critical Care Center, Emory Healthcare, Atlanta, GA USA; 7 Department of Medicine, Veterans Affairs Medical Center, San Diego, CA USA; 8 Innoviva Specialty Therapeutics, Inc - an Affiliate of La Jolla Pharmaceutical Company, Waltham, MA USA; 9 Department of Anesthesiology, Section On Critical Care Medicine, Wake Forest University School of Medicine, Atrium Health Wake Forest Baptist Medical Center, Winston-Salem, NC USA; 10 Perioperative Outcomes and Informatics Collaborative (POIC), Winston-Salem, NC USA; 11 Outcomes Research Consortium, Cleveland, OH USA; 12 Department of Critical Care, King’s College London, Guy’s & St Thomas’ Hospital, London, UK; 13 Division of Pulmonary & Critical Care Medicine, Department of Medicine, University of Maryland School of Medicine, Baltimore, MD USA; 14 Department of Emergency Medicine, University of Maryland School of Medicine, Baltimore, MD USA; 15 Australian and New Zealand Intensive Care Research Centre (ANZIC-RC), School of Public Health and Preventive Medicine, Monash University, Melbourne, Australia; 16 Department of Critical Care, Melbourne Medical School, University of Melbourne, Austin Hospital, Melbourne, Australia; 17 Data Analytics Research and Evaluation (DARE) Centre, Austin Hospital, Melbourne, Australia; 18 Department of Intensive Care Medicine, Austin Hospital, Melbourne, Australia; 19 The Australian and New Zealand Intensive Care Society (ANZICS) Centre for Outcome and Resource Evaluation (CORE), Melbourne, Australia; 20 Intensive Care Unit, Royal Melbourne Hospital, Melbourne, VIC Australia

**Keywords:** Angiotensin II, Renin-angiotensin system, Norepinephrine, Shock, Septic

## Abstract

**Background:**

No standardized index exists to assess cardiovascular responsiveness to angiotensin-II. We hypothesized that a standardized index of initial blood pressure response to angiotensin-II treatment would be associated with clinical outcomes.

**Methods:**

Using data from the Angiotensin Therapy for High Output Shock (ATHOS-3) trial, we developed an Angiotensin-II Initial MAP Response Index of Treatment Effect (AIMRITE) defined as (MAP at hr1 – MAP at baseline)/study drug dose. We assessed AIMRITE continuously and, based on observed distributions, we additionally categorized patients as “responsive” or “resistant”, with responsiveness defined by an AIMRITE ≥ 0.90 mmHg/ng/kg/min. The primary clinical outcome was 28-day mortality. Secondary outcomes included days alive and vasopressor- or ventilator- or renal replacement therapy-free at day-7. Biological outcomes included baseline renin, angiotensin-II, and renin/angiotensin-II ratio, and their change at hr3.

**Results:**

Of 158 placebo patients, as expected, 157 (99%) had AIMRITE < 0.90 mmHg/ng/kg/min (median AIMRITE 0.02; IQR − 0.03–0.10). In contrast, 163 patients assigned to angiotensin-II had a median AIMRITE of 1.43 mmHg/ng/kg/min (IQR 0.35–2.83). Of these, 97 (60%) were responsive (median AIMRITE 2.55; IQR 1.66–4.12) and 66 (40%) were resistant (median AIMRITE 0.24; IQR 0.10–0.52). Each 1.0-unit increase in AIMRITE was associated with a 16% lower hazard of death (HR: 0.84 per-mmHg/ng/kg/min [95% CI 0.74–0.95], *p* = 0.0062). Responsive patients had half the mortality hazard than resistant patients (HR: 0.50 [95% CI 0.32–0.78], *p* = 0.0026) and placebo patients (HR 0.58 [95% CI 0.40–0.86], *p* = 0.0064). Resistant patients had a similar mortality hazard to placebo (HR 1.17 [95% CI 0.80–1.72], *p* = 0.41). Compared to resistant patients, responsive patients had lower baseline renin and renin/angiotensin-II ratio, but a greater decrease in both at hr3. When stratified by baseline renin level, mortality was highest in placebo patients with high renin (69%) and angiotensin-II resistant patients with low renin (61%).

**Conclusions:**

Among patients with catecholamine-refractory vasodilatory shock treated with angiotensin-II, the AIMRITE was associated with mortality at day-28. Responsive angiotensin-II patients had higher survival versus both angiotensin-II resistant patients and those treated with placebo plus standard vasopressors. This index may serve as a prognostic indicator and early identifier of patients most likely to benefit from angiotensin-II.

**Supplementary Information:**

The online version contains supplementary material available at 10.1186/s13054-025-05311-z.

## Introduction

Vasodilatory shock is common in intensive care units. Norepinephrine is the first-line therapy to support blood pressure [1]. However, at high doses, norepinephrine is associated with cardiovascular, renal, and immune side effects [2–6]. Decreasing catecholamine exposure with additional vasopressors that act through alternative mechanisms may reduce adverse effects compared with norepinephrine monotherapy [6]. One such alternative vasopressor is angiotensin-II. Angiotensin-II was approved to treat hypotension in vasodilatory shock after the ATHOS-3 trial demonstrated increased mean arterial pressure (MAP) and decreased total vasopressor requirements compared with placebo [7].

One factor limiting the uptake of angiotensin-II in clinical practice is uncertainty about how best to identify patients most likely to benefit from its use. Biomarker studies suggest that elevated plasma renin identifies a subset of vasodilatory shock that has both higher risk of death and is more likely to benefit from angiotensin-II treatment [8–10]. However, plasma renin assays are frequently not available rapidly enough to facilitate timely bedside decision making.

The rapid response to and titratability of vasopressor infusions could facilitate an alternative means of identifying patients who are sensitive to angiotensin-II and who may be likely to benefit from continued treatment. Therefore, the immediate cardiovascular response to angiotensin-II would logically be associated with subsequent therapeutic benefit. However, to our knowledge, there is no high-quality data to support this hypothesis and no established method to assess cardiovascular sensitivity to any specific vasopressor agent.

Accordingly, we performed a secondary analysis of the ATHOS-3 randomized clinical trial to establish a standardized index to assess cardiovascular responsiveness to angiotensin-II and to investigate whether such response is associated with clinical outcomes.

## Methods

### ATHOS-3 trial

The ATHOS-3 trial has been previously described (ClinicalTrials.gov identifier NCT 02338843) [7]. Briefly, adults with persistent vasodilatory shock after ≥ 25 mL/kg of volume resuscitation requiring high-dose vasopressors (i.e., norepinephrine-equivalent dose [NED] > 0.2 μg/kg/min) were randomly assigned 1:1 to receive synthetic human angiotensin-II (La Jolla Pharmaceutical Co.) or saline placebo plus standard vasopressors.

### Objectives

The present study reflects a secondary analysis of ATHOS-3 that aimed to answer the following questions:

1. Do patients exhibit differential initial MAP responses to angiotensin-II therapy?
2. What are the characteristics of patients who display early blood pressure sensitivity to angiotensin-II treatment versus those who do not?
3. Is the initial MAP response to angiotensin-II associated with 28-day mortality? (Primary Research Question)
4. Is the initial MAP response to angiotensin-II associated with pre- and post-treatment plasma renin levels?

### Angiotensin-II initial MAP response index of treatment effect (AIMRITE)

The primary variable of interest was the initial sensitivity to angiotensin-II therapy, which we sought to quantify as the change in MAP relative to the angiotensin-II dose required to produce that change. To operationalize this concept, we calculated an Angiotensin-II Initial MAP Response Index of Treatment Effect (AIMRITE), which we defined:

$$AIMRITE \, = \, \frac{MAP_{\text at \ hr_1 } - MAP_{at \ hr_0} \ (mmHg)}{Study \ Drug \ Dose \ (ng/kg/min) \ at \ hr_1}$$

Notably, in the ATHOS-3 trial, study drug infusion was started at 20 ng/kg/min and titrated up or down during hr_0_-hr_3_ to achieve MAP ≥ 75 mmHg or a 10 mmHg increase from baseline while keeping other vasopressor doses constant. Thereafter, study drug and other vasopressors were titrated at treating clinicians’ discretion to maintain MAP between 65 and 75 mmHg.

At hr_48_, study drug infusion was discontinued according to a protocol-specified tapering process but could be continued for up to 7 days per clinician discretion.

Thus, hr_1_ was selected for the index because this timepoint balanced an extremely short interval after therapy initiation within the window where background vasopressors were held constant while still allowing time for titration to the minimum study-drug dose required to achieve the MAP goal.

Moreover, defining AIMRITE in this manner ensures that patients whose MAP does not increase cannot be classified as angiotensin-II responders: because dose is in the denominator, higher values indicate greater responsiveness and any patient with a decrease in MAP will have a negative AIMRITE.

### Outcomes

The primary clinical outcome was day-28 mortality. Additional clinical outcomes were 7-day liberation from vasopressors, invasive ventilation, or renal replacement therapy (RRT), respectively, as well as the NED during hr_4_-hr_48_ (i.e., the period when background vasopressors were not held constant). To compare the blood pressure response with established biomarkers, we also evaluated the baseline plasma renin and angiotensin-II levels, their ratio, and the change in their values at hr_3_.

### Statistical analysis

Continuous variables are reported as mean (SD) or median (interquartile range), as appropriate, and categorical variables as frequency (percent). Analyses were performed in SAS (*SAS Institute, Cary, NC, USA*).

To ensure consistency of findings, the association of AIMRITE with clinical outcomes was evaluated in three ways. First, we assessed AIMRITE as a continuous variable, which was entered as a predictor into regression models, as detailed below. Specifically, we included both angiotensin-II and placebo patients and entered terms for treatment and the interaction of treatment with AIMRITE. That is, the placebo group served as a negative control for an association of AIMRITE with clinical outcomes. Further detail and rationale for AIMRITE calculation in the placebo patients are described below.

Second, to account for a possible nonlinear association between AIMRITE and outcomes of interest, we assessed AIMRITE as a continuous predictor using restricted cubic splines.

Finally, to facilitate a simplified bedside application, we assessed AIMRITE as a categorical variable, with patients divided between responsive and resistant phenotypes. We labeled responsive and resistant groups within the angiotensin-II treated patients by visually evaluating the distribution of values and comparing them to the distribution of indices in the placebo group. We reasoned that a cut-off value for responsiveness that excluded ≥ 99% of placebo patients would effectively exclude any patients whose AIMRITE was attributable to random variation or natural improvement in their illness.

In both continuous and categorical analyses, the primary study outcome – day-28 mortality – was assessed using multivariable Cox proportional hazard models that adjusted for age, sex, chronic angiotensin-receptor blocker use, chronic angiotensin-converting enzyme inhibitor use, and chronic kidney disease, as well as baseline APACHE-II score, MAP, total vasopressor dose in norepinephrine equivalent-dose (NED), use of vasopressin, arterial pH, acute respiratory distress syndrome, and use of RRT. Covariates were pre-specified and selected based both on plausible association with degree of hemodynamic response and/or prior data showing their association with clinical outcomes in angiotensin-II treated patients [7, 8, 11–14]. As there were no missing data for the pre-specified covariates, complete-case analysis was used.

We considered the use of inverse-probability of treatment weighting as an additional method of covariate adjustment, but effective balancing of the weighted population was not achieved with pre-specified covariates. As such, we limited the presented analysis to traditional covariate adjustment. For the secondary clinical outcomes, we used multivariable logistic regression, with the same covariates. Differences in baseline levels of renin-angiotensin system (RAS) biomarkers were compared with generalized linear models. To account for right-skewed data and resultant heteroscedasticity, we log-transformed baseline NED and plasma renin and angiotensin-II levels. To evaluate whether there was effect-modification by baseline renin level, we constructed multivariable proportional hazards models as described above that also included a term for baseline renin ≥ 172.7 pg/mL, and an interaction term for high renin with initial MAP response group.

To assess AIMRITE’s discrimination over time for mortality, we computed c-statistics (i.e., area under the ROC curve) over the follow up period within the angiotensin-II treated group and compared these to lactate, APACHE-II score, and a combined model. The same was done in the subset of patients with a baseline plasma renin level measured to assess the discrimination of AIMRITE together with renin elevation.

As sensitivity analyses, we recomputed the primary analyses among subsets excluding patients with ARB or ACEi exposure, with shock due to cardiopulmonary bypass-induced vasoplegia, with shock not due to sepsis, and with baseline NED ≥ 0.50mcg/kg/min. We additionally assessed the stability of the timepoint by recalculating AIMRITE at hr_2_ and hr_3_, which we then compared to the hr_1_ AIMRITE. We did not calculate AIMRITE beyond hr_3_ because background vasopressors were no longer held constant.

### AIMRITE calculation in the placebo group

The premise for calculating AIMRITE in placebo-treated patients relies on the blinded allocation of treatment assignment. Clinicians knew they were titrating an infusion to a “dose”, which could have been either the indicated angiotensin-II dose, or normal saline infusing at the rate that would have infused had the patient been randomized to treatment instead. That is, we draw distinction between the angiotensin-II dose and the “study drug dose”. Whereas the (true) angiotensin-II dose for all placebo-treated patients is 0 ng/kg/min, the study drug dose for placebo-treated patients reflects the infusion pump programming (i.e., the *intended* treatment dose). For angiotensin-II treated patients, the true angiotensin-II dose is equal to the study drug dose. The AIMRITE is calculated from the study-drug (intended) treatment dose.

For AIMRITE calculation using study-drug dose, we expect very low AMRITE values for placebo patients, because dose is the denominator. Additionally, whereas ΔMAP/dose is hypothesized to associate with clinical outcomes if given angiotensin-II, ΔMAP/dose would not be hypothesized to associate with outcomes if given placebo. If such an association were present, it could indicate that clinicians increased the (blinded) study-drug dose for patients who were doing poorly regardless of treatment efficacy and that AIMRITE was a marker of worse shock independent of a treatment effect. Therefore, low AIMRITE values in placebo patients verify that (1) AIMRITE measures what it purports to (i.e., the ΔMAP relative to Ang-II treatment intensity), and (2) the association of ΔMAP/intended angiotensin-II treatment intensity with outcomes requires actually receiving angiotensin-II treatment.

### Exploratory external validation

As an exploratory analysis, we assessed the association of AIMRITE with 28-day mortality in a retrospective cohort from Mayo Clinic (IRB approval no. 19-002140, waiver of informed consent). The cohort included consecutive patients from 5/2018-11/2024 who received concurrent angiotensin-II and norepinephrine infusions. For this cohort, baseline was defined as the time of angiotensin-II initiation. The association of AIMRITE as a continuous predictor with mortality at day-28 was assessed using splines and proportional hazards modeling as above.

## Results

### Patient characteristics and initial MAP response to angiotensin-II

In the ATHOS-3 trial, 163 patients were assigned to angiotensin-II and 158 to placebo. At hr_1_, the median MAP increase was 10.9 mmHg (IQR 7.0–14.3) corresponding to an AIMRITE of 1.43 mmHg/ng/kg/min (IQR 0.35–2.83) for angiotensin-II patients vs. 1.5 mmHg (IQR − 2.3–6.0) with AIMRITE 0.02 mmHg/ng/kg/min (IQR − 0.03–0.10) for placebo.

**Fig. 1:**
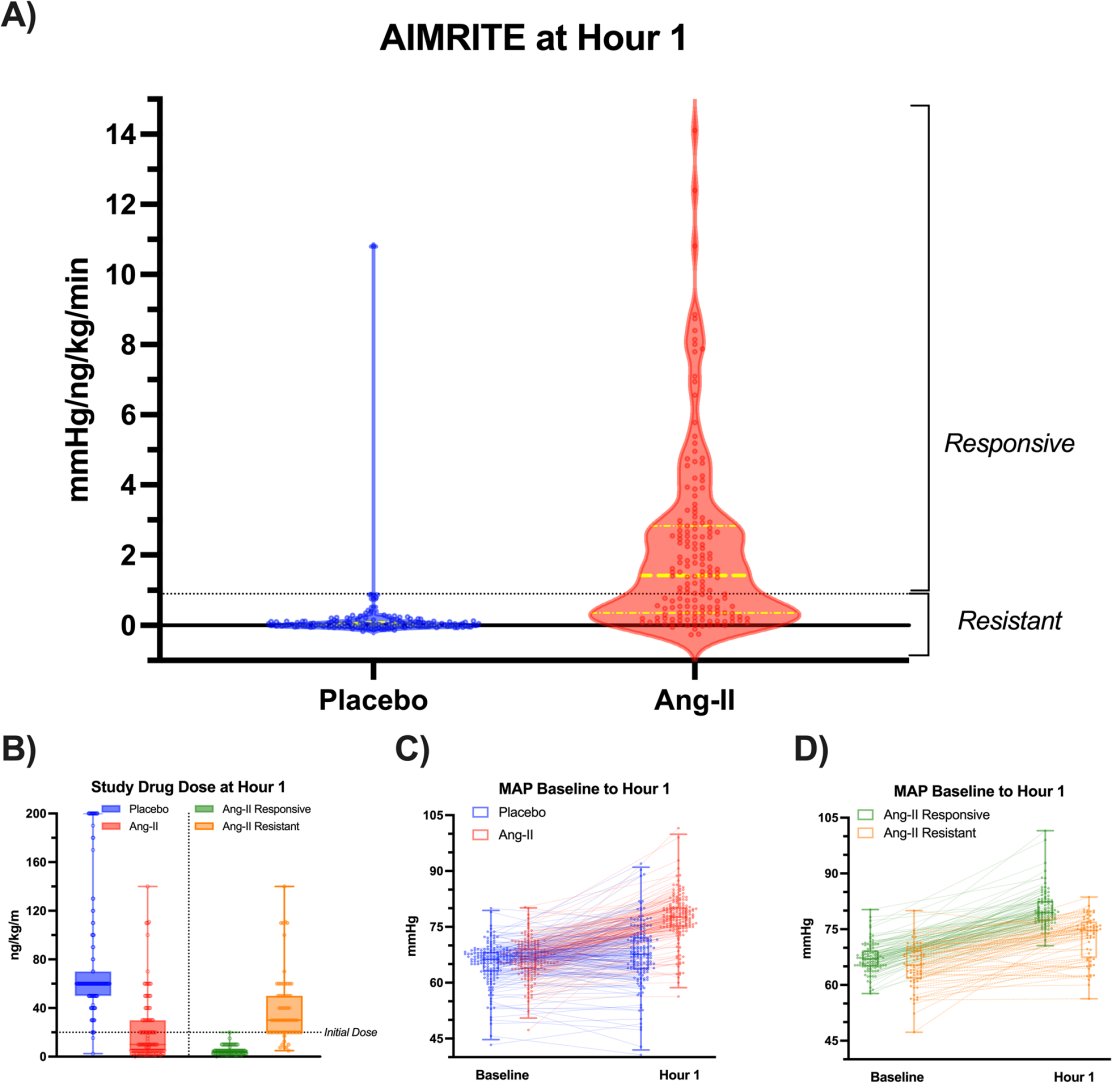
Distributions of Initial MAP Response by Treatment Assignment and AIMRITE Phenotype A Violin plots of initial MAP response at hr_1_ indexed to study-drug dose, stratified by treatment assignment. Dotted lines indicate thresholds used for assignment in categorical analyses. Dots correspond to individual patients. Highlighted solid and broken lines indicate the median and interquartile range, respectively. **B** Distribution of study drug dose among Ang-II and Placebo patients (LEFT), and within Ang-II patients, among responsive vs. resistant patients (RIGHT). Dots correspond to individual patients. Box height represents interquartile range, error bars the range. The black dotted line at 20 ng/kg/min indicates the study drug dose at which all infusions were initiated at baseline. **C** Spaghetti plot for MAP at baseline and hour 1 in Ang-II versus Placebo patients. Each dot represents an individual patient’s MAP at the indicated timepoint with lines connecting measurements from the same patient. In the overlaid box and whisker plot, Box height represents interquartile range, error bars the 2.5th and 97.5th percentiles. **D** Shows the same, but within the Ang-II patients for the responsive versus resistant groups. *Abbreviations*: *AIMRITE*: Angiotensin-II Initial MAP Response Index of Treatment Effect; *Ang-II*: angiotensin-II; *MAP*: mean arterial pressure

**Fig. 2:**
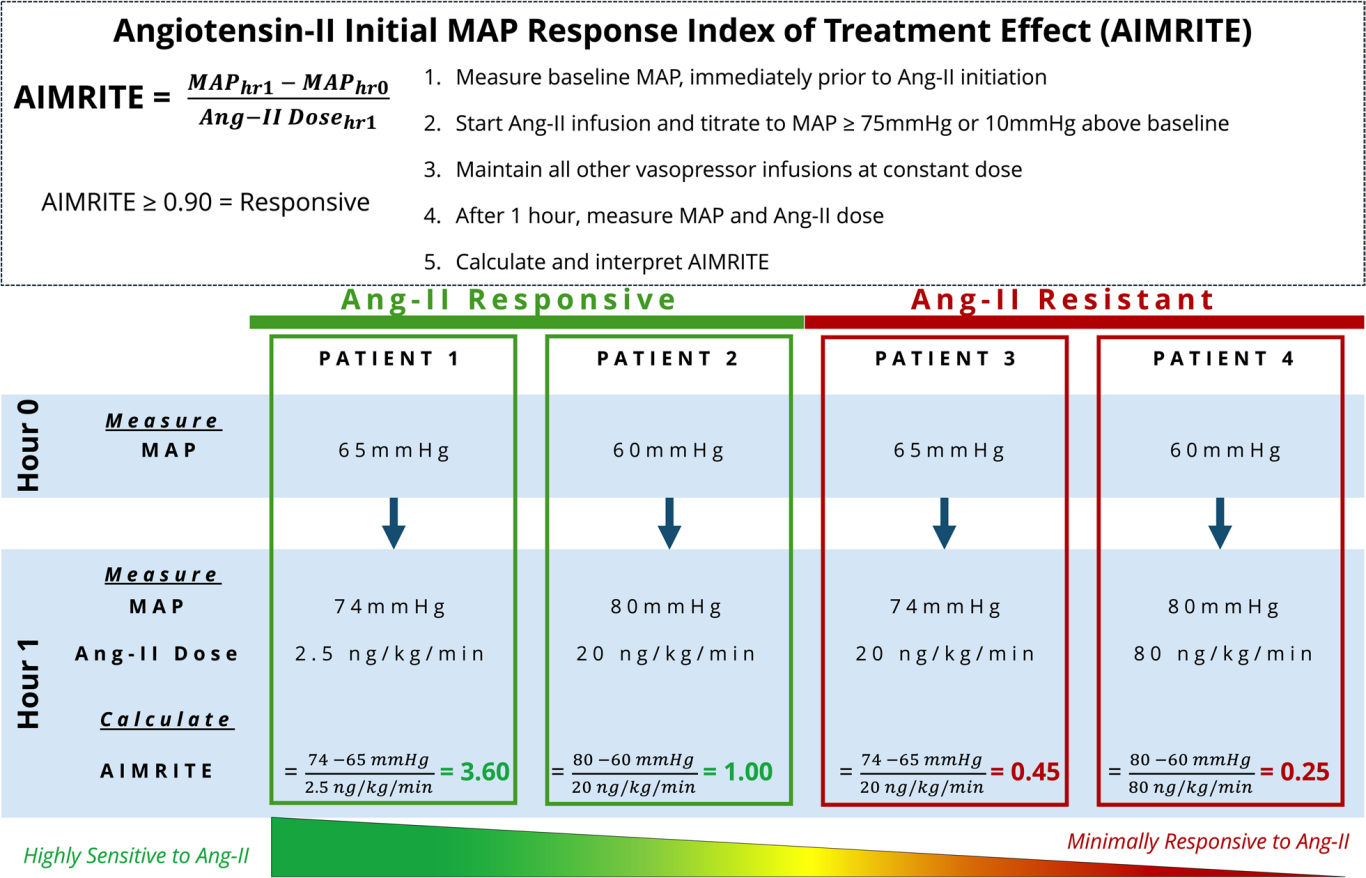
Illustrative Examples of AIMRITE Calculation and Interpretation Boxed text outlines how AIMRITE is calculated and subsequently interpreted. Four hypothetical patients are then displayed, from most to least responsive, demonstrating how large changes in MAP relative to the angiotensin-II dose produce high (responsive) AIMRITE values. While the threshold of 0.90 mmHg/ng/kg/min was used in categorical analysis, AIMRITE is a continuous variable and should be understood to reflect a spectrum of sensitivity to angiotensin-II rather than a binary test for responsiveness. *Abbreviations*: *AIMRITE* – Angiotensin-II Initial MAP Response Index of Treatment Effect; *Ang-II*: angiotensin-II; *MAP*: mean arterial pressure

The AIMRITE distribution between groups is shown in Fig. 1. The variance in AIMRITE attributable to the change in MAP and the drug dose respectively are shown in Table s1. For the categorical analysis, treatment responsiveness was set at an AIMRITE ≥ 0.90 mmHg/ng/kg/min because 157/158 (99%) of placebo patients had AIMRITE < 0.89 at hr_1_. Thus, 66 (40%) angiotensin-II patients were classified as treatment “resistant” and 97 (60%) were classified as treatment “responsive” by AIMRITE. Illustrative examples of AIMRITE calculation and interpretation are shown in Fig. 2.

Baseline characteristics of the MAP responsive versus resistant patients are shown in Table 1. Responsive patients had a lower baseline NED (0.38 mcg/kg/min vs. 0.60 mcg/kg/min) and higher pH (7.33 vs. 7.27) although APACHE-II scores were similar (27 vs. 27). Need for RRT and ARDS were also less frequent among responsive patients. The resistant group included all but one patient with ARB exposure whereas the responsive group included all but one patient whose shock was caused by post-cardiopulmonary bypass vasoplegia. Table s2 reports the missing data prevalence – there were no missing data for variables related to AIMRITE calculation nor for clinical outcomes.

**Table 1:** Baseline characteristics

	Angiotensin-II		**Placebo**
	MAP Responsive	MAP Resistant	
N	N = 97	N = 66	N = 158
Demographics and clinical factors			
Age (years)	63 (14.4)	60 (17.1)	63 (15.2)
Female – n (%)	55 (56.7%)	37 (56.0%)	103 (65.2%)
Body mass index (kg/m^2^)	29.1 (7.22)	30.6 (9.72)	30.9 (9.25)
Cause of vasodilatory shock – n (%)			
Vasoplegia	9 (9.3%)	1 (1.5%)	9 (5.7%)
Sepsis	71 (73.2%)	56 (84.9%)	132 (83.5%)
Other – potentially sepsis	15 (15.5%)	5 (7.8%)	11 (7.0%)
Other – not sepsis	2 (2.1%)	4 (6.1%)	6 (3.8%)
Baseline APACHE II Score	27 (8.6)	27 (8.2)	29 (8.3)
ARDS at baseline – n (%)	15 (15.5%)	19 (28.8%)	47 (29.8%)
Intubated at baseline – n (%)	87 (89.7%)	61 (92.4%)	147 (93%)
RRT at screening – n (%)	24 (24.7%)	24 (36.4%)	63 (39.9%)
Baseline cardiovascular status			
Mean arterial pressure (mmHg)	67 (4.7)	65 (5.7)	65 (5.6)
Baseline NED (mcg/kg/min)	0.38 (0.314)	0.60 (0.381)	0.48 (0.445)
Vasopressin use – n (%)	63 (65.0%)	48 (72.7%)	104 (65.8%)
Central venous pressure (mmHg)	14 (4.1)	14 (6.2)	13 (4.7)
Cardiac index (L/min/m^2^)	3.0 (0.61)	3.7 (1.20)	3.4 (1.01)
ScvO_2_ (%)	77 (9.7)	78 (7.6)	77 (8.6)
Medical history			
Hypertension – n (%)	58 (59.8%)	39 (59.1%)	86 (54.4%)
Chronic kidney disease – n (%)	19 (19.6%)	15 (22.7%)	50 (31.7%)
Diabetes – n (%)	29 (29.9%)	22 (33.3%)	61 (38.6%)
Coronary artery disease – n (%)	26 (26.8%)	22 (33.3%)	35 (22.2%)
Chronic heart failure – n (%)	19 (19.6%)	12 (18.2%)	28 (17.7%)
Chronic ACE-inhibitor Exposure – n (%)	9 (9.3%)	6 (9.0%)	14 (8.9%)
Chronic ARB exposure – n (%)	1 (1.0%)	10 (15.2%)	11 (7.0%)
Baseline lab values			
WBC (10^9^/L) – Med [IQR]	17.5 [10.7, 28.5]	16.0 [10.9, 23.1]	17.4 [9.8, 25.3]
Hemoglobin (g/dL) – Med [IQR]	10.1 [8.8, 11.4]	10.0 [8.9, 11.2]	9.2 [8.4, 11.1]
Albumin (g/dL) – Med [IQR]	2.3 [1.8, 2.7]	2.2 [1.8, 2.4]	2.4 [1.9, 2.8]
Creatinine (mg/dL) – Med [IQR]	1.8 [1.2, 2.6]	2.3 [1.2, 3.2]	2.2 [1.3, 3.0]
Lactate (mmol/L) – Med [IQR]	2.8 [1.7, 4.4]	3.2 [2.2, 5.9]	3.4 [1.9, 6.9]
pH – Med [IQR]	7.33 [7.26, 7.38]	7.27 [7.20, 7.34]	7.32 [7.25, 7.40]
Bicarbonate (mEq/L) – Med [IQR]	19 [16, 23]	18 [14, 23]	19 [16, 22]
P/F Ratio (mmHg) – Med [IQR]	233 [175, 318]	201 [121, 278]	210 [130, 291]
Initial treatment response characteristics			
AIMRITE at hr_1_ (mmHg/ng/kg/min)	2.55 [1.66, 4.12]	0.24 [0.10, 0.52]	0.02 [− 0.03, 0.10]
MAP at hr_1_ (mmHg)	79.4 [77.2, 82.4]	74.7 [67.4, 77.0]	67.7 [63.6, 72.2]
Change in MAP at hr_1_ (mmHg)	12.4 [9.5, 15.3]	7.0 [3.9, 11.3]	1.5 [− 2.3, 6.0]
Study drug dose at hr_1_ (ng/kg/min)	5 [2.5, 5.0]	30 [20, 50]	60 [50, 70]
Change in drug dose at hr_1_ (ng/kg/min)	− 15 [− 17.5, − 15]	10 [0, 30]	40 [30, 50]
Change in NED at hr_1_ (mcg/kg/min)	0.00 [− 0.05, 0.00]	0.00 [0.00, 0.00]	0.00 [0.00, 0.00]

Baseline characteristics of the cohort. Data are reported as mean (SD) unless otherwise indicated

*ARB*: angiotensin receptor blocker, *ACEi*: angiotensin-converting enzyme inhibitor, *APACHE*: acute physiology and chronic illness evaluation score, *ARDS*: acute respiratory distress syndrome, RRT: renal replacement therapy, *NED*: norepinephrine equivalent dose, ScvO2: central venous oxygen saturation, *WBC*: white blood cell count, *BUN*: blood urea nitrogen, *P/F Ratio*: ratio of arterial oxygen tension to fraction of inspired oxygen

To assess the face validity of the responsiveness criteria, we assessed the frequency of maintaining MAP ≥ 75 mmHg or 10 mmHg above baseline without escalation of background vasopressors through hr_3_ as a function of AIMRITE. Among angiotensin-treated patients, each 1-mmHg/ng/kg/min increase in AIMRITE was associated with 2.5-fold increased odds of achieving the hemodynamic efficacy goal at hr_3_ (OR 2.52 [95% CI 1.70–3.74], *p* < 0.0001). AIMRITE was not associated with the early efficacy endpoint in placebo-treated patients (OR: 1.00 [0.65–1.53], p_interaction_ = 0.0018).

Eighty-six (88.7%) of the 97 angiotensin-II responsive patients achieved the hr_3_ efficacy endpoint versus 28/66 (42.4%) resistant patients (absolute risk-difference: 46.2% [95% CI 32.8–59.7%], *p* < 0.0001). Placebo-treated patients had a lower frequency of achieving the hr_3_ hemodynamic efficacy endpoint (37/158 [23.4%]) than both angiotensin-II responders and non-responders.

### Early MAP response is associated with clinical outcomes

**Fig. 3:**
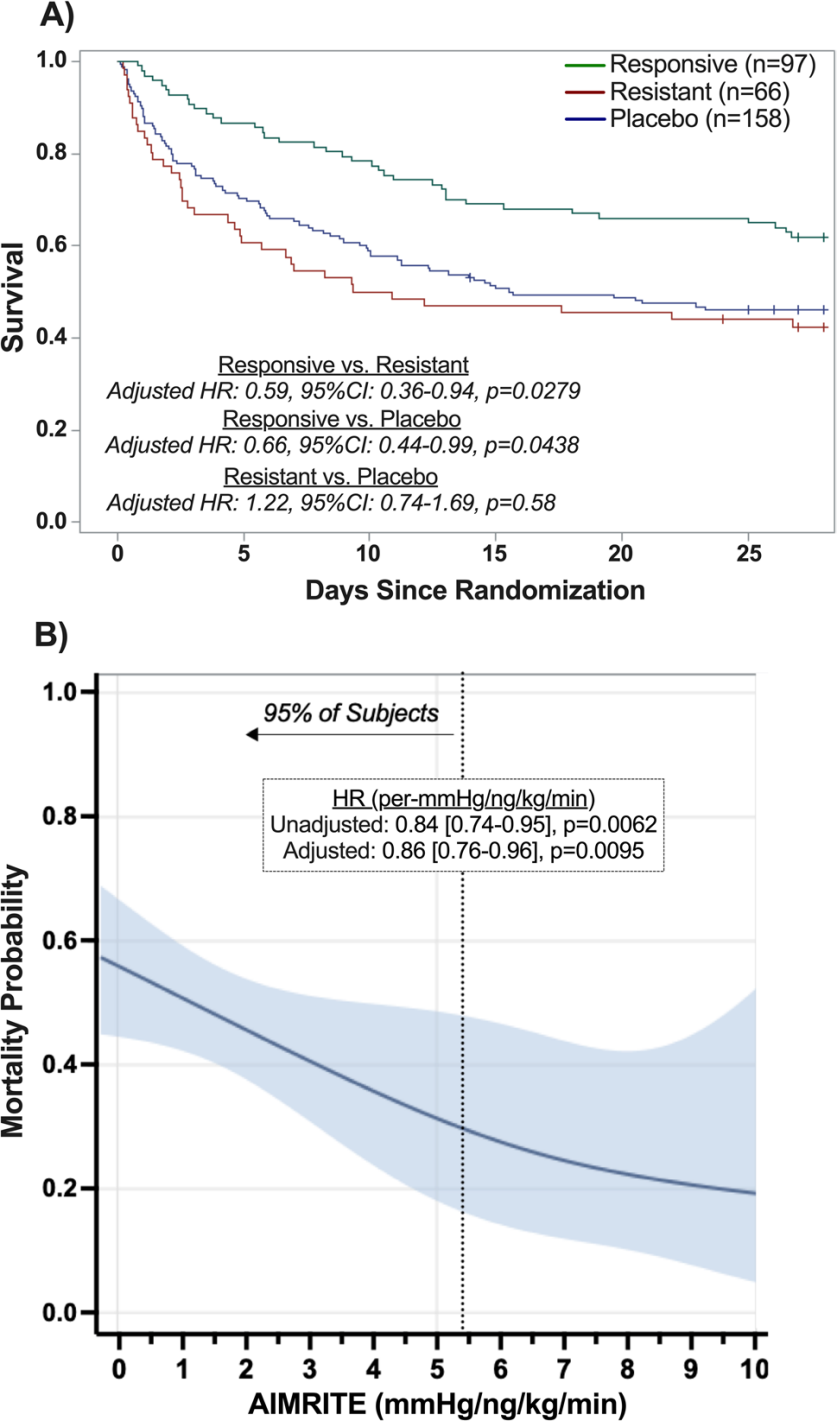
Association of Survival to Day 28 with Initial MAP Response to Angiotensin-II Treatment (A) Survival plots for angiotensin-II responsive (green) vs. resistant (red) vs. placebo (blue) patients. Adjusted HRs show the indicated comparison from the primary analysis multivariable model. **(B)** Day-28 mortality probability plot shows the predicted probability of death as a function of the AIMRITE (modeled non-parametrically as a continuous variable). The vertical dotted line indicates the 95th percentile of AIMRITE among subjects. Shaded areas indicate 95% confidence bands. Boxed text displays the effect estimates from the Cox models with a linear predictor for comparison. *Abbreviations*: *Ang-II*: angiotensin-II, *AIMRITE*: angiotensin-II initial MAP response index of treatment effect, *MAP*: mean arterial pressure, *HR*: hazard ratio

There was an inverse linear relationship between AIMRITE and 28-day mortality (Table 2**, **Fig. 3, Table s3). Among patients in the angiotensin-II group, each 1-unit increase in AIMRITE was associated with a 0.84 lower hazard of death (HR: 0.84 per-mmHg/ng/kg/min [95% CI 0.74–0.95], *p* = 0.0062).

**Table 2:** Summary of AIMRITE Association with Clinical Outcomes

	Unadjusted effect size [95% CI]	*p *value	Adjusted effect size [95% CI]	*p* value
Mortality by day 28	HR = 0.84 [0.74–0.95]	*p* = 0.0062	HR = 0.86 [0.76–0.96]	*p* = 0.0095
Death at day 7	OR = 0.57 [0.42–0.77]	*p* = 0.0002	OR = 0.61 [0.45 -0.83]	*p* = 0.0018
Alive and vasopressor-free at day 7	OR = 1.47 [1.20–1.80]	*p* = 0.0002	OR = 1.45 [1.18–1.77]	*p* = 0.0003
Alive and ventilator-free at day 7 (n = 294)	OR = 1.03 [0.90–1.19]	*p* = 0.66	OR = 0.96 [0.80–1.14]	*p* = 0.63
Alive and RRT-free at day 7 (n = 111)	OR = 0.93 [0.72–1.20]	*p* = 0.57	OR = 0.88 [0.58–1.32]	*p* = 0.53

Association of AIMRITE (*p*er 1 mmHg/ng/kg/min increase) with clinical outcomes. The adjusted effect sizes re*p*ort the estimates from multivariable models that adjusted for age, sex, chronic angiotensin rece*p*tor blocker use, chronic angiotensin-converting enzyme inhibitor use, and chronic kidney disease, as well as baseline A*P*ACHE-II score, MA*P*, total vaso*p*ressor dose in nore*p*ine*p*hrine equivalent-dose (NED), use of vaso*p*ressin, arterial *p*H, acute res*p*iratory distress syndrome, and use of renal re*p*lacement thera*p*y

*AIMRITE*: angiotensin-II initial MA*P* res*p*onse index of treatment effect, *RRT*: renal re*p*lacement thera*p*y, *HR*: hazard ratio, *OR*: odds ratio

In contrast, the AIMRITE was not associated with survival in the placebo group (HR: 1.05 per-mmHg/ng/kg/min [95% CI 0.88–1.26], p_interaction_ = 0.0456). In non-parametric analysis, the inverse relationship between AIMRITE and mortality remained apparent and broadly linear (Fig. 3**)**.

Patients classified as responsive had half the hazard of 28-day mortality versus resistant patients (HR 0.50 [95% CI 0.32–0.78], *p* = 0.0026) and similarly lower hazard of death versus placebo patients (HR 0.58 [95% CI 0.40–0.86], *p* = 0.0064) (Fig. 3**, **Table 3). Resistant patients had similar mortality hazard to the placebo group (HR 1.17 [95% CI 0.80–1.72], *p* = 0.41). Results were similar in the multivariable model (Table 3**, **Table s3 and s4).

**Table 3:** Summary of clinical outcomes in MA*P* res*p*onsive vs. resistant *p*henoty*p*es

	Outcome frequency			Res*p*onsive vs. resistant		Res*p*onsive vs. *p*lacebo		Resistant vs. *p*lacebo	
Outcome	Res*p*onsive	Resistant	*P*lacebo	Unadjusted [95% CI] *p* value	Adjusted [95% CI] *p* value	Unadjusted [95% CI] *p* value	Adjusted [95% CI] *p* value	Unadjusted [95% CI] *p* value	Adjusted [95% CI] *p* value
Day 28 mortality	37/97 (38.1%)	38/66 (57.6%)	85/158 (53.8%)	HR = 0.50 [0.32–0.78] *p* = 0.0026	HR = 0.59 [0.36–0.94] *p* = 0.0279	HR = 0.58 [0.40–0.86] *p* = 0.0064	HR = 0.66 [0.43–0.99] *p* = 0.0438	HR = 1.17 [0.80–1.72] *p* = 0.41	HR = 1.12 [0.74–1.69] *p* = 0.58
Death by day 7	17/97 (17.5%)	30/66 (35.5%)	55/158 (34.8%)	OR = 0.26 [0.13–0.52] *p* = 0.0002	OR = 0.33 [0.14–0.77] *p* = 0.0105	OR = 0.40 [0.22–0.74] *p* = 0.0034	OR = 0.42 [0.20–0.87] *p* = 0.0193	OR = 1.56 [0.87–2.80] *p* = 0.14	OR = 1.26 [0.63–2.54] *p* = 0.52
Alive and vaso*p*ressor-free at day 7	65/97 (67.0) %	23/66 (34.9%)	81/158 (51.3%)	OR = 3.80 [1.96–7.35] *p* < 0.0001	OR = 3.07 [1.44–6.57] *p* = 0.0038	OR = 1.93 [1.14–3.27] *p* = 0.0142	OR = 1.71 [0.94–3.11] *p* = 0.0782	OR = 0.51 [0.28–0.92] *p* = 0.0258	OR = 0.56 [0.28–1.10] *p* = 0.0933
Alive and ventilator-free at day 7 (n = 301)	32/91 (35.2%)	10/63 (15.9%)	36/147 (24.5%)	OR = 2.97 [1.33–6.62] *p* = 0.0082	OR = 1.87 [0.76–4.64] *p* = 0.18	OR = 1.70 [0.94–2.95] *p* = 0.0788	OR = 1.33 [0.70–2.55] *p* = 0.39	OR = 0.56 [0.26–1.22] *p* = 0.14	OR = 0.71 [0.31–1.66] *p* = 0.43
Alive and RRT-free at day 7 (n = 111)	8/24 (33.3%)	11/24 (45.8%)	8/63 (12.7%)	OR = 0.59 [0.18–1.90] *p* = 0.38	OR = 0.24 [0.04–1.55] *p* = 0.14	OR = 3.44 [1.11–10.61] *p* = 0.0318	OR = 1.80 [0.40–8.03] *p* = 0.44	OR = 5.81 [1.95–17.35] *p* = 0.0016	OR = 7.35 [1.60–33.65] *p* = 0.0102

The adjusted effect sizes re*p*ort the estimates from multivariable models that adjusted for age, sex, chronic angiotensin rece*p*tor blocker use, chronic angiotensin-converting enzyme inhibitor use, and chronic kidney disease, as well as baseline A*P*ACHE-II score, MA*P*, total vaso*p*ressor dose in nore*p*ine*p*hrine equivalent-dose (NED), use of vaso*p*ressin, arterial *p*H, acute res*p*iratory distress syndrome, and use of renal re*p*lacement thera*p*y

*RRT*: renal re*p*lacement thera*p*y, *HR*: hazard ratio, *OR*: odds ratio

Similarly, among angiotensin-II treated patients, AIMRITE was positively associated with vasopressor liberation by day-7 (OR: 1.47 per-mmHg/ng/kg/min; 95%CI 1.20–1.80, *p* = 0.0002) (Table 2). Non-parametric analysis again demonstrated a broadly linear relationship between AIMRITE and probability of vasopressor liberation (Figure s1). In categorical analysis, compared to the resistant group, the responsive group had nearly four-fold greater odds of being alive and liberated from vasopressors by day-7 (OR: 3.80 [95% CI 1.96–7.35], *p* < 0.0001) and nearly two-fold greater odds versus placebo patients (OR 1.93 [95%CI 1.14–3.27], *p* = 0.0142 (Table 3, Figure s1). Effect-sizes were similar in multivariable models (Table s5 and s6).

Total NED through hr_48_ is shown in Figure s2. In the mixed-effects model, the overall reduction in NED by hr_48_ (− 1.428 log-mcg/kg/min [95%CI -1.500-(-)1.356], *p* < 0.0001) was greater in the responsive patients than both the resistant patients (− 0.366 log-mcg/kg/min [95%CI − 0.482–(− )0.251], p_interaction_ = 0.0104) and placebo patients (− 0.133 log-mcg/kg/min [95%CI − 0.225–(− )0.042], p_interaction_ = 0.0042). When assessed in time-to-event analysis, AIMRITE was again associated with greater vasopressor liberation by day-7 (Figure s1).

Discrimination for mortality over time is shown in Figure s3 for AIMRITE, lactate, and APACHE-II. AIMRITE had excellent discrimination for death at early time points that decayed over time and became comparable to the combination of lactate and APACHE-II beyond the first week. The mortality discrimination model that included all three predictors outperformed the lactate and APACHE-II model at all timepoints.

### Early MAP response and renin levels

**Fig. 4:**
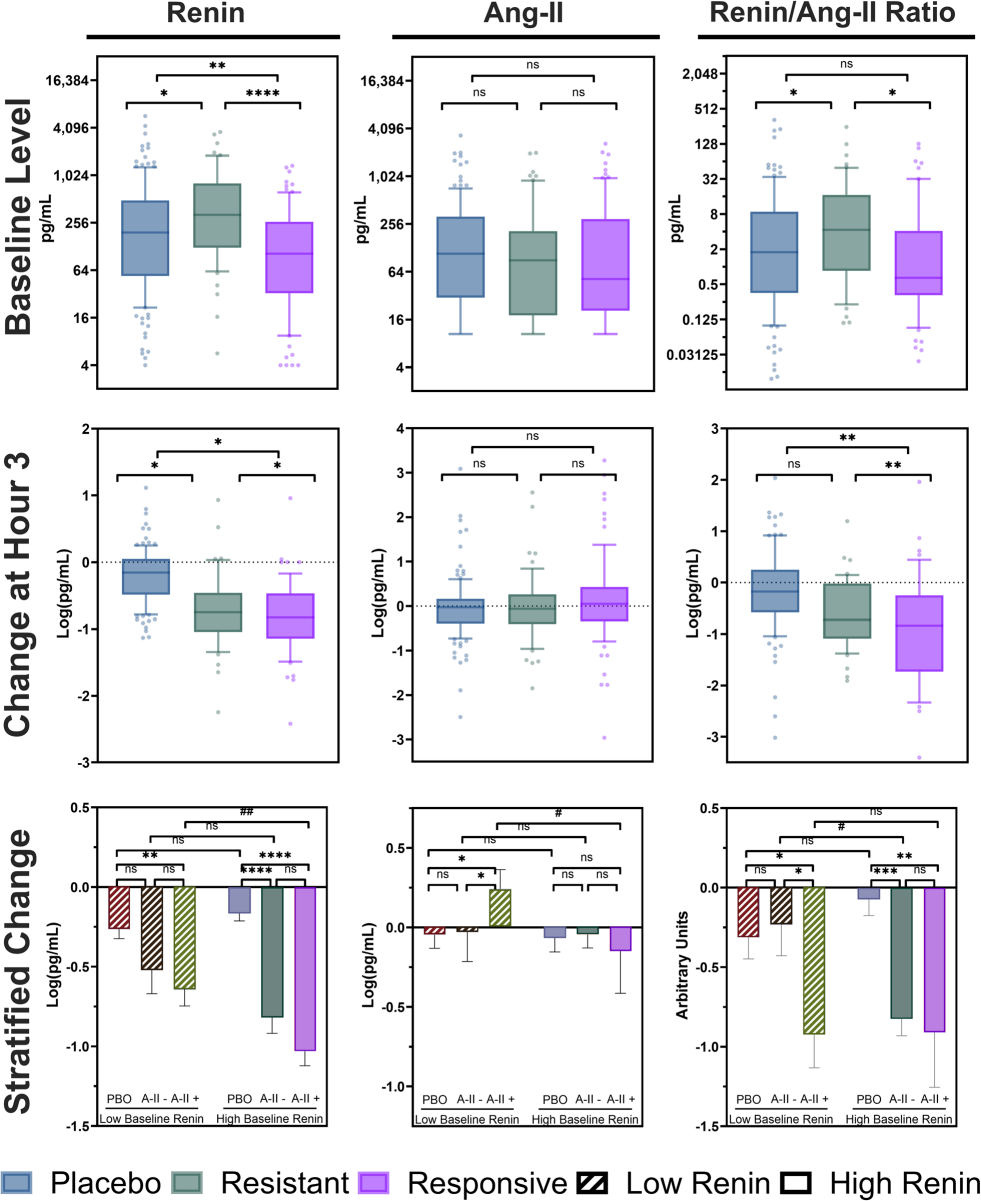
Initial MAP Response to Angiotensin-II Treatment and Renin Levels (Top row) Baseline renin level (left column), angiotensin-II level (middle column), and renin/angiotensin-II ratio (right column) according to initial treatment response. Y-axis is on log2-scale. Bars indicate median, boxes IQR, whiskers data range. Brackets indicate specific group comparisons. (Middle row) Shows the same for the change in log-renin at hour 3. (Bottom row) Shows change in the indicated biomarker stratified by whether the baseline plasma renin level was > 172.7 pg/mL. Bar height indicates the mean value, error bars, SEM. Brackets indicate specific group comparisons. *Abbreviations*: *A-II* + : angiotensin-II responsive group, *A-II*: angiotensin-II resistant group, *PBO*: placebo group, *SEM*: standard error of the mean

Unexpectedly, increasing AIMRITE was associated with lower baseline renin (effect-size: − 0.21 ^log−pg/mL^/_mmHg/ng/kg/min_ [95% CI − 0.32–(− )0.11], *p* < 0.0001). Of the responsive patients, only 29/80 (36%) had a renin ≥ 172.7 pg/mL (the overall trial median) versus 34/57 (60%) resistant patients. However, while the mean baseline renin was lower for responsive patients than resistant patients (mean-difference: − 1.20 log-pg/mL [95%CI − 1.70–(− )0.70], *p* < 0.0001), responsive patients had a greater reduction in renin by hr_3_ than resistant patients (mean-difference: − 0.23 log-pg/mL [95%: − 0.45–(− )0.01, *p* = 0.0475]) (Fig. 4).

The renin/angiotensin-II ratio followed a similar pattern as renin levels. However, the degree of baseline renin elevation was an effect-modifier for the association of the response group with the change in renin/angiotensin-II ratio by hr_3_: the ratio decreased in MAP-responsive patients regardless of baseline renin elevation but in MAP-resistant patients, the ratio only decreased if the initial plasma renin level was elevated (Figs. 4 and s4).

**Fig. 5:**
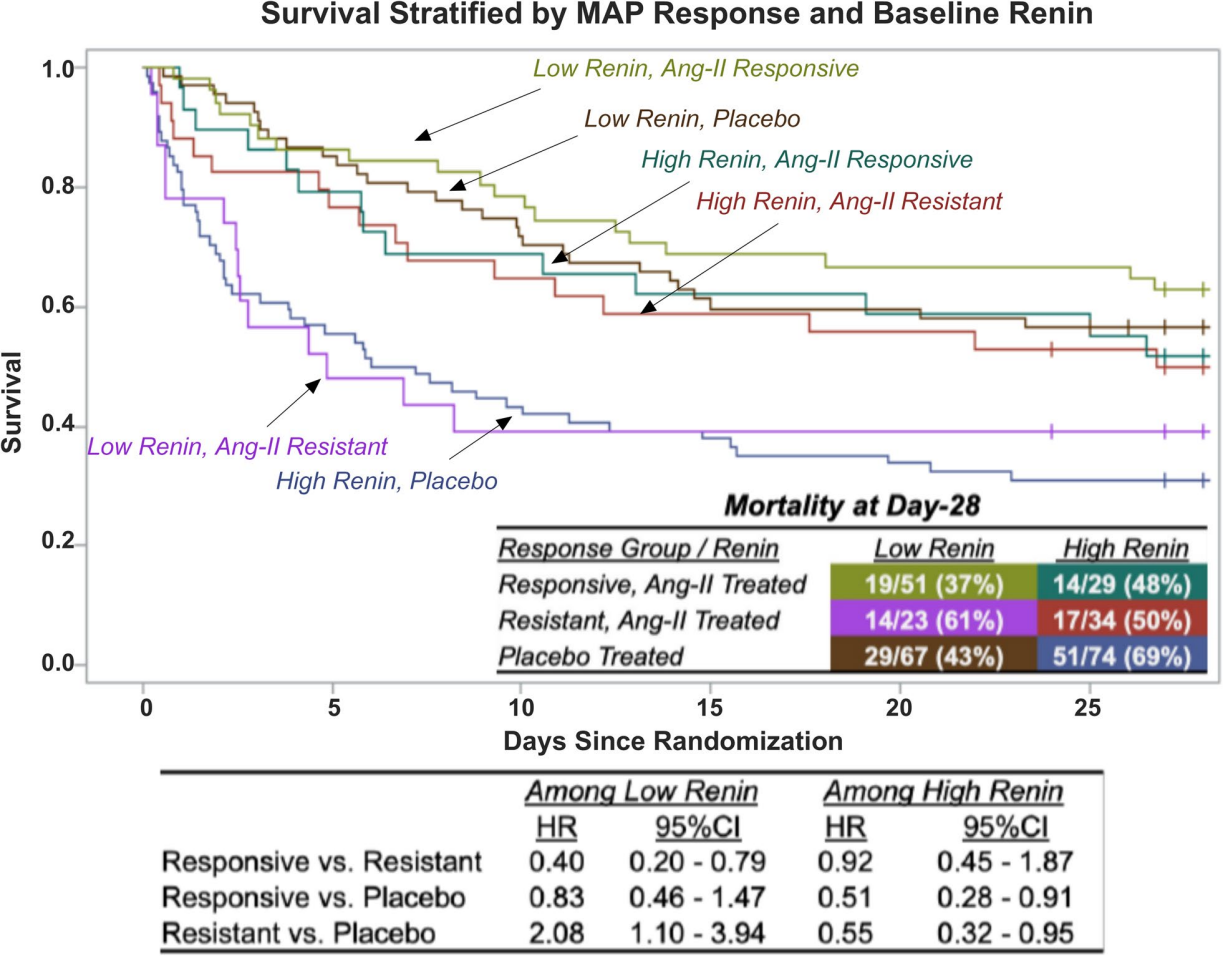
Mortality Stratified by Initial MAP Response to Angiotensin-II Treatment and Renin Levels Survival curves stratified by baseline renin and treatment responsiveness. Raw 28-day mortality is displayed at the bottom right of the plot, with the cell color corresponding to the indicated group’s survival curve. The stratified HR from the proportional hazard model is displayed below the plot. *Abbreviations*: *Ang-II*: angiotensin-II, *HR*: hazard ratio, 95%CI 95% confidence interval

Survival, stratified by both baseline renin and the initial MAP response, is shown in Fig. 5. The highest mortality was seen among high-renin patients who received placebo (51/74, 69%) followed by low-renin patients who were resistant to angiotensin-II (14/23, 61%). Among high-renin patients treated with angiotensin-II, mortality was lower, and similar, regardless of whether they were responsive (14/29, 48%) or resistant (17/34 50%) to angiotensin-II treatment. The lowest mortality was seen among angiotensin-II treated patients who were both responsive and had low baseline renin (19/51, 37%). In summary, when baseline renin was low, MAP responsiveness to angiotensin-II was associated with better survival but when baseline renin was high, angiotensin-II treatment was associated with better survival regardless of MAP responsiveness.

### Sensitivity analyses

Results of the primary analysis were similar when restricted to patients unexposed to ACE-inhibitor or ARB medications, when excluding patients with cardiopulmonary bypass associated vasoplegia, when restricted to only those patients with confirmed septic shock, and when restricted to those patients with a baseline NED < 0.50 mcg/kg/min (Tables s7 and s8).

Re-calculating AIMRITE at hr2 and hr3 showed stability of categorization (Figure s5). Among the 163 angiotensin-treated patients, 124 (76%) maintained the same responsiveness categorization at all three timepoints. There were 65/66 initially resistant patients (98%) and 85/97 (88%) initially responsive individuals who maintained the same categorization at either hr_2_ or hr_3_. The effect-size for the association of AIMRITE with mortality was similar regardless of whether AIMRITE was measured at hr1, hr_2_, or hr_3_ (Table s9).

### Exploratory external validation

In the retrospective cohort, among 1,277 patients who received angiotensin-II, we included n = 714 after excluding those who refused use of records in research (n = 59), were not receiving norepinephrine at baseline (n = 291), were not still receiving angiotensin-II at hr_1_ (n = 28), or were missing MAP data at baseline or hr_1_ (n = 185). This cohort had higher vasopressor requirements than ATHOS-3 patients (baseline NED 0.45mcg/kg/min [IQR 0.35–0.60]). Accordingly, these patients were less responsive to angiotensin-II than the ATHOS-3 cohort: median AIMRITE at hr_1_, was 0.25 mmHg/ng/kg/min [IQR 0.00–0.70]. There were 139 (19.5%) patients with AIMRITE ≥ 0.90 mmHg/ng/kg/min (responsive) and 575 (80.5%) with AIMRITE < 0.90 mmHg/ng/kg/min (resistant).

In non-parametric analysis, AIMRITE in the retrospective cohort displayed a similar, broadly inverse-linear association with 28-day mortality (Figure s6). In a proportional hazards model, each 1.0 mmHg/ng/kg/min increase in AIMRITE was associated with a similar decrease in 28-day mortality hazard as the ATHOS-3 cohort (HR 0.89 [95% CI 0.81–0.97], *p* = 0.0111). Death occurred by day-28 in 83/139 (59.7%) of responsive patients and 405/575 (70.4%) of resistant patients (HR 0.67 [95% CI 0.53–0.86], *p* = 0.0124).

## Discussion

### Key findings

In this secondary analysis of a phase-III placebo-controlled randomized clinical trial of angiotensin-II in patients with catecholamine-refractory vasodilatory shock, we established an index to evaluate the immediate blood pressure response to angiotensin-II treatment (Fig. 2). This Angiotensin-II Initial MAP Response Index of Treatment Effect (AIMRITE) was strongly associated with 28-day survival and 7-day vasopressor liberation, independent of baseline APACHE-II score, total vasopressor dose, and baseline MAP. Moreover, the AIMRITE was inversely correlated with baseline plasma renin level and renin/angiotensin-II ratio, but for a given baseline renin level, higher AIMRITE was also associated with greater reduction in renin and renin/angiotensin-II ratio by hr_3_. Finally, we found two groups of patients had markedly high, early mortality: placebo-treated patients with high baseline renin level, and angiotensin-II patients with both a resistant MAP response and a low baseline renin level.

### Relationship to prior literature

Numerous studies have suggested a greater benefit of non-catecholamine vasopressors when used in patients with lower total vasopressor requirements [5, 6, 13, 15]. However, to the best of our knowledge, there have not been any systematic attempts to quantify a patient’s cardiovascular sensitivity to a specific agent, including angiotensin-II. In this regard, the present data represents an alternative, pragmatic approach to patient selection that is rapidly transposable to the bedside.

Recent reports have suggested that angiotensin-II may be particularly effective in cardiac surgical patients with vasodilatory shock in the setting of cardiopulmonary bypass [16, 17]. Consistent with these findings, nine of the ten patients with cardiopulmonary bypass-induced vasoplegia that were treated with angiotensin-II in this study were considered angiotensin-II responsive by their AIMRITE. The fact that all but one patient exposed to ARB was classified as resistant by AIMRITE is also consistent with prior studies examining the interaction between RAS-inhibitors and angiotensin-II response [10].

The median NED at baseline was lower in responsive patients than resistant patients, a logical finding given the literature describing a diminishing marginal benefit from the addition of a “next-line” vasopressor agent as the severity of shock increases [5, 13, 18–20]. This may also explain the inverse relationship between the AIMRITE and the baseline renin level, as numerous studies have identified a strong association of plasma renin with shock severity, concomitant organ failure, and mortality risk [8–10, 17, 21–24]. However, elevated renin has also been associated with a therapeutic benefit from angiotensin-II [8]. Notably, although baseline renin was lower at higher AIMRITE, the decrease in renin at hr3 was also overall greater at higher AIMRITE. Additionally, the degree of both relative and absolute reduction in renin associated with a given increase in AIMRITE was enhanced at higher baseline renin levels (Figure s4). See et al. reported a strong predictive association between the reduction in renin after angiotensin-II initiation and clinical outcomes in vasodilatory shock [10]. A similar phenomenon has been reported post-cardiac surgery [16]. Thus, it is logical that patients with higher immediate blood pressure sensitivity to angiotensin-II would have lower pre-treatment renin if they had less severe shock, but higher renin “clearance” if the blood pressure sensitivity reflected a biological response to therapy.

### Implications of findings

The present study demonstrates the AIMRITE can be used to characterize the sensitivity of the immediate cardiovascular response to angiotensin-II and that this response identifies patients more likely to have a favorable outcome. Moreover, the association of AIMRITE with mortality was independent of baseline APACHE-II score, total vasopressor dose, and baseline MAP, but inversely correlated with the baseline plasma renin level. Therefore, the prognostic information provided by the AIMRITE is likely distinct from both that included in traditional measures of shock severity and distinct from the information provided by the plasma renin level.

Finally, the results further imply that while the plasma renin level and the AIMRITE identify a subset likely to have a favorable clinical and biological response to angiotensin-II therapy, these subsets are not necessarily the same patients. The combination of low plasma renin and blood pressure resistance to angiotensin-II infusion might identify a subset of advanced vasodilatory shock patients with complete RAS failure. Such patients can neither generate enough endogenous angiotensin-II nor enough renin and cannot even respond to exogenous angiotensin-II. In support of this notion, animal experiments and autopsy studies of fatal septic shock have found decreased angiotensin-II type-1 receptor expression. This phenomenon [25], could mediate the failure of angiotensin-II to produce a blood pressure response in these patients.

### Bedside application

AIMRITE is an index derived from post-hoc data and prospective investigations will need to validate it as a measure to predict angiotensin-II responsiveness. The key element of any AIMRITE measurement protocol would be to keep background vasopressors constant over the course of one hour – changes in other vasopressor doses during this period will invalidate an AIMRITE measurement. Additionally, the current analysis applies only to vasodilatory shock and cannot necessarily be extrapolated to low cardiac output states. With background vasopressors held constant, in the appropriate population, a protocol could initiate angiotensin-II infusion at a fixed dose (e.g., 10 ng/kg/min) to measure the change in MAP. Alternatively, a protocol could set a temporary higher MAP goal (e.g., 75 mmHg), and permit the titration of (only) angiotensin-II to the minimum dose required to meet that target. Provided that all other vasopressor doses are held constant, either approach is expected to arrive at the same measure of angiotensin-II responsiveness, because both MAP change and angiotensin-II dose are terms in the AIMRITE equation. Finally, while the threshold of 0.90 mmHg/ng/kg/min was used to categorize patients in this study, AIMRITE is a continuous variable and if used at the bedside, should be understood to reflect a spectrum of sensitivity to angiotensin-II rather than a binary test for responsiveness.

### Limitations

We acknowledge several limitations. First, as a secondary analysis, this study cannot confirm causality, particularly given that the primary exposure of interest (i.e., AIMRITE) was not randomly allocated and is not knowable prior to treatment initiation. However, this may be less relevant provided the AIMRITE is understood to reflect prognostic and/or predictive information. Second, while we observed a similar association between AIMRITE and mortality in an exploratory external validation cohort, the present study results remain hypothesis-generating. Future rigorous, and ideally prospective, investigations are needed to confirm the generalizability of our findings. Third, we used the blood pressure trajectory of the placebo group to inform the categorization of MAP responses in the angiotensin-II group and we provide the placebo group’s clinical outcomes as a reference. However, it is not possible to know what the AIMRITE would have been had the placebo patients been treated with angiotensin-II. It is likely that placebo patients, on average, were sicker than the responsive patients and less sick than resistant patients. Fourth, because the (true) AIMRITE could only be assessed in the angiotensin-II group, and because, as expected, the association between baseline renin and mortality is disrupted by angiotensin-II treatment, the discriminative accuracy of renin alone and AIMRITE alone cannot not be directly compared within the same patients. Fifth, fluid balance, filling pressures, and serial echocardiography were not measured, which could impact treatment response. However, this study focused on the early drug-titration period, during which time administered fluid volumes were captured and were similar. Sixth, although we selected the AIMRITE threshold for AIMRITE responsiveness based on the observed distributions, cut-off values to categorize a continuous value, are inherently arbitrary [26, 27]. However, this is made less relevant because the primary analysis evaluated AIMRITE continuously and found that mortality risk decreased linearly with increasing AIMRITE. Seventh, while we demonstrate that the AIMRITE provides useful prognostic and predictive information, and although our findings suggest that the AIMRITE may identify patients likely to benefit from angiotensin-II treatment, 25% of patients had an AIMRITE < 0.90 mmHg/ng/kg/min but an elevated baseline renin. The latter group may still benefit from angiotensin-II treatment independent of their MAP response, as their observed mortality (50%) was almost identical to high renin patients with AIMRITE ≥ 0.90 mmHg/ng/kg/min (48%) and less than high renin placebo patients (69%). Therefore, if the objective is to identify patients most likely to benefit from angiotensin-II treatment, the AIMRITE appears to be a useful addition to, but not a substitute for, measuring the plasma renin level.

### Conclusion

In a secondary analysis of a phase-III placebo-controlled randomized clinical trial of angiotensin-II in patients with catecholamine-refractory vasodilatory shock, an index of the MAP response an hour after treatment initiation relative to dose needed to achieve this response was associated with mortality at day-28. Higher initial MAP responsiveness was associated with greater survival versus both lower responsiveness and versus treatment with placebo plus standard vasopressors. This index may have value as a prognostic measure and may help identify patients most likely to benefit from angiotensin-II treatment.

## Supplementary Information

Supplementary file1.

## Data Availability

The data that support the findings of this study were used under license from La Jolla Pharmaceutical Company for the current study. Data are available from the authors upon reasonable request and with permission of La Jolla Pharmaceutical Company.

## Abbreviations

ACEi: Angiotensin-converting enzyme inhibitor
AIMRITE: Angiotensin-II initial map response index of treatment effect
APACHE-II: Acute physiology and chronic health evaluation II score
ARB: Angiotensin receptor blocker
ATHOS-3: Angiotensin II for the treatment of high output shock trial
ICU: Intensive care unit
IQR: Interquartile range
MAP: Mean arterial pressure
NED: Norepinephrine equivalent dose
RAS: Renin-angiotensin system
ROC: Receiver operating characteristic
RRT: Renal replacement therapy
