# Supplementary material for: An index of the initial blood pressure response to angiotensin II treatment and its association with clinical outcomes in vasodilatory shock
Source: Crit Care. 2025 Feb 19;29:81. doi: 10.1186/s13054-025-05311-z (PMC11837372; doi:10.1186/s13054-025-05311-z)
Supplement: Supplementary file 1 — Supplementary file1. [file 13054_2025_5311_MOESM1_ESM.docx]

**SUPPLEMENTARY MATERIAL**

Title: An Index of the Initial Blood Pressure Response to Angiotensin II Treatment and its Association with Clinical Outcomes in Vasodilatory Shock

Authors: Daniel E. Leisman, MD, MSCR *et al.* on behalf of the ATHOS-3 Investigators.

| **Table of Contents:** |  |
| --- | --- |
| Figure-s1: Norepinephrine Equivalent Dose Over Time | Pg. 2 |
| Figure-s2: Association of Vasopressor Liberation by Day 7 with AIMRITE | Pg. 3 |
| Figure-s3: Comparative Discrimination for Mortality over Time | Pg. 5 |
| Figure-s4: Change in Renin at Hour 3 as a Function of AIMRITE and Baseline Renin | Pg. 6 |
| Figure-s5: Alluvial Plot of AIMRITE Categorization Over the First 3 Hours | Pg. 7 |
| Figure-s6: Association of AIMRITE with 28-Day Mortality in an Independent Cohort | Pg. 8 |
| Table-s1: Explained Variance in AIMRITE from Component Variables | Pg. 9 |
| Table-s2: Missing Data Prevalence for Key Fields | Pg. 10 |
| Table-s3: Multivariable Model - Association of AIMRITE with 28-Day Mortality | Pg. 11 |
| Table-s4: Multivariable Model - Association of AIMRITE Category with 28-Day Mortality | Pg. 12 |
| Table-s5: Multivariable Model - Association of AIMRITE with Alive & Vasopressor-Free at Day-7 | Pg. 13 |
| Table-s6: Multivariable Model - Association of AIMRITE Category with Alive & Vasopressor-Free at Day-7 | Pg. 14 |
| Table-s7: Sensitivity Analyses for Association of AIMRITE with Mortality at Day-28 | Pg. 15 |
| Table-s8: Sensitivity Analyses for Association of AIMRITE Response Categories with Mortality at Day-28 | Pg. 16 |
| Table-s9: Serial Calculation of AIMRITE Over First 3 Hours and Association with 28-Day Mortality | Pg. 17 |

**SUPPLEMENTAL FIGURES**

**
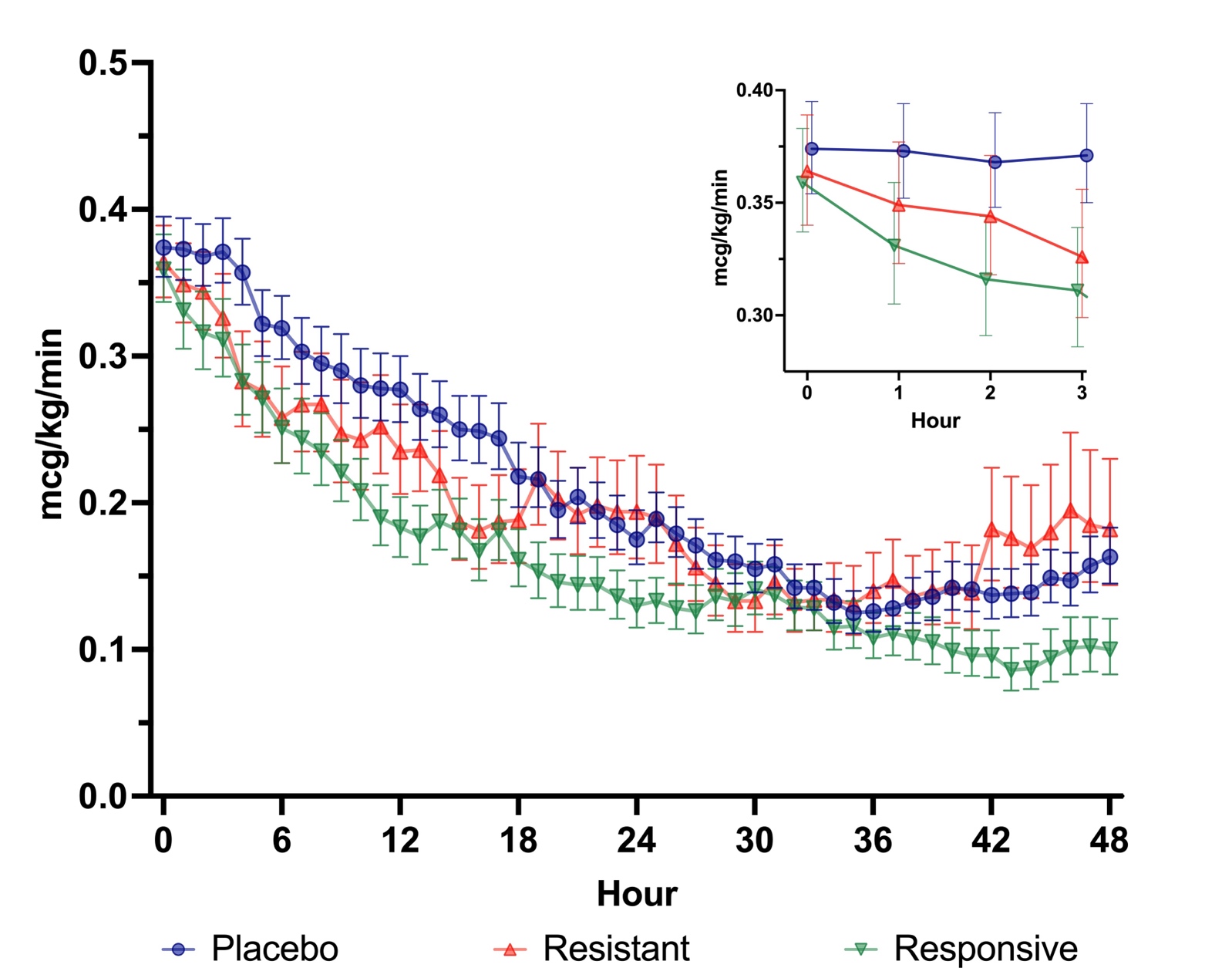
**

**Figure-s1: Norepinephrine Equivalent Dose Over Time**

Plot shows the hourly NED by response group over the first 48 hours of treatment. Markers and error bars indicate the geometric mean and standard error of the indicated group.

**
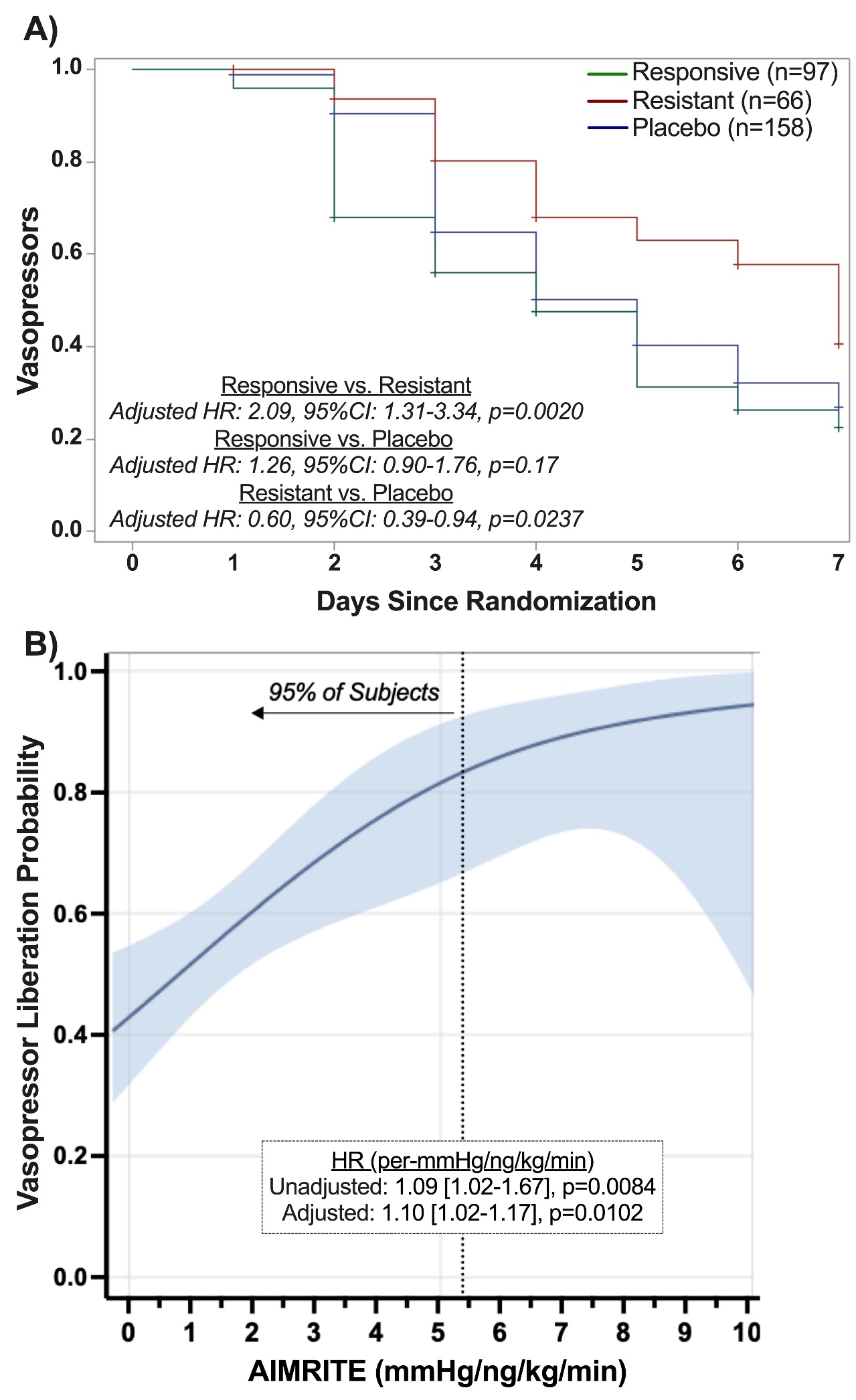
**

**Figure-s2: Association of Vasopressor Liberation by Day 7 with Initial MAP Response to Angiotensin-II Treatment**

**(A)** Survival curves for angiotensin-II responsive (green) vs. resistant (red) vs. placebo (blue) patients. The ‘event’ in the survival plot is vasopressor liberation. Adjusted HRs show the indicated comparison from the primary analysis multivariable model. **(B)** Day-7 vasopressor probability plot shows the predicted probability of death as a function of the AIMRITE (modeled non-parametrically as a continuous variable). The vertical dotted line indicates the 95^th^ percentile of AIMRITE among subjects. Shaded areas indicate 95% confidence bands. Boxed text displays the effect estimates from the Cox models with a linear predictor for comparison. Abbreviations: Ang-II – angiotensin-II, AIMRITE – angiotensin-II initial MAP response index of treatment effect, MAP – mean arterial pressure, HR – hazard ratio.

**
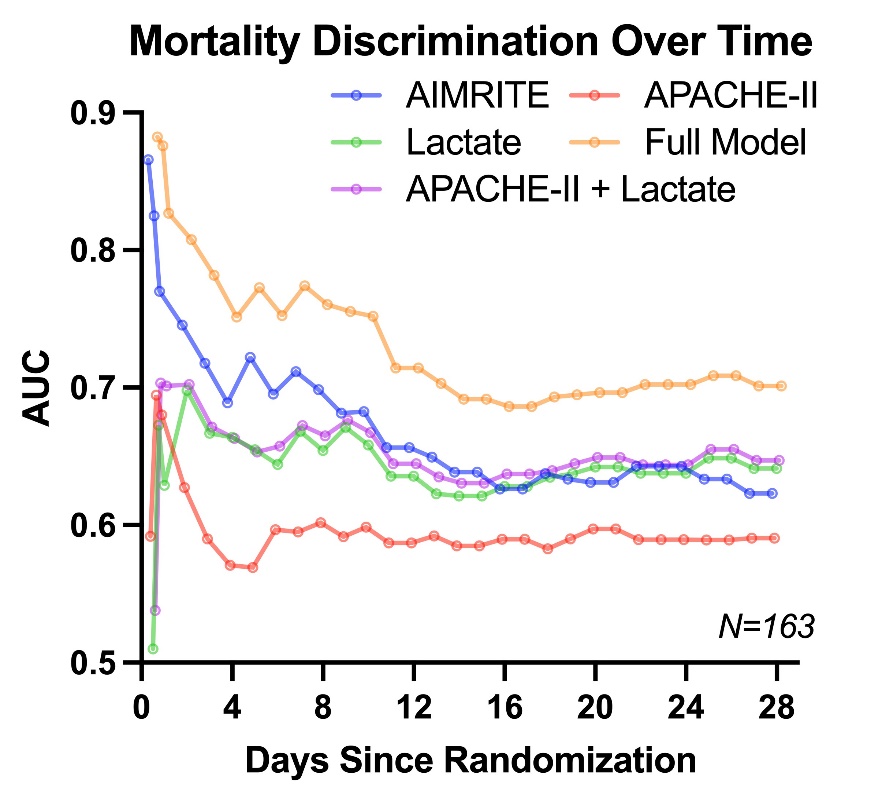
**

**Figure-s3: Comparative Discrimination for Mortality over Time**

Plot of the area under the ROC curve for mortality (y-axis) by the indicated (x-axis) for AIMRITE, APACHE-II score, and baseline lactate among patients in the angiotensin-II group. The baseline lactate was imputed for the n=21 patients in the angiotensin-II group who were missing these data. The curve labeled “APACHE-II + Lactate” refers to a model that included both variables, while “Full Model” refers to a model that included AIMRITE, APACHE-II score, and baseline lactate. Abbreviations: AIMRITE – Angiotensin-II Initial MAP Response Index of Treatment Effect; AUC – area under the curve; APACHE-II – acute physiology and chronic health evaluation-II; ROC curve – receiver operating characteristic curve.

**
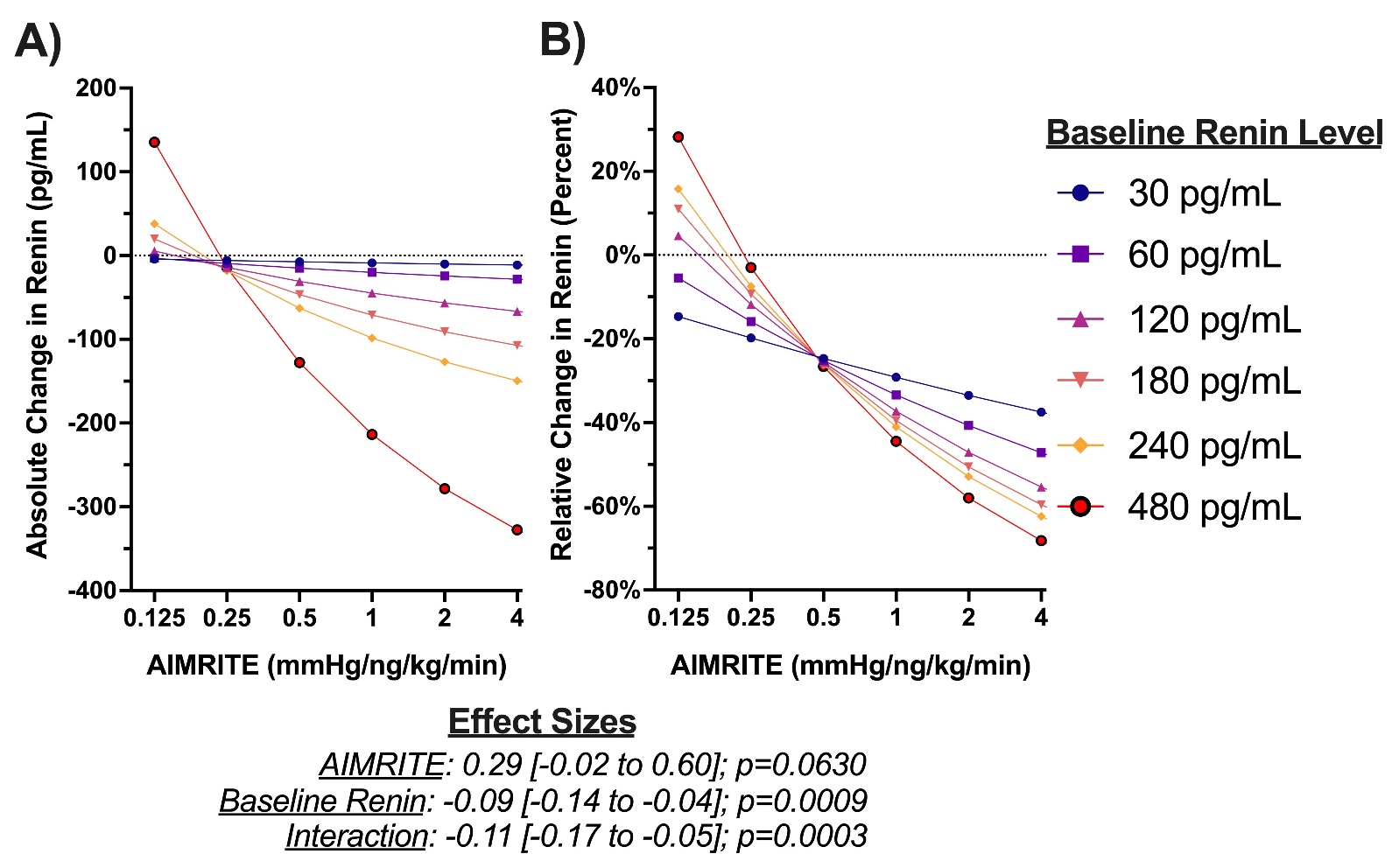
**

**Figure-s4: Continuously Modeled Change in Renin at Hour 3 as a Function of AIMRITE and Baseline Renin**

**(A)** Least-squares change in renin (y-axis) as a function of AIMRITE (x-axis). Each curve corresponds to a different baseline renin level, as displayed in the legend at right. The model estimates used to produce these curves are displayed below the graph. The model’s intercept was -0.0467. All terms used in the model were Ln-transformed but the untransformed are back-calculated and shown in the figure. **(B)** Displays the same, but the y-axis shows the renin at hour 3 as percent change from the baseline value. Curves were produced from the same model as (A).

Abbreviations: AIMRITE – Angiotensin-II Initial MAP Response Index of Treatment Effect.

**
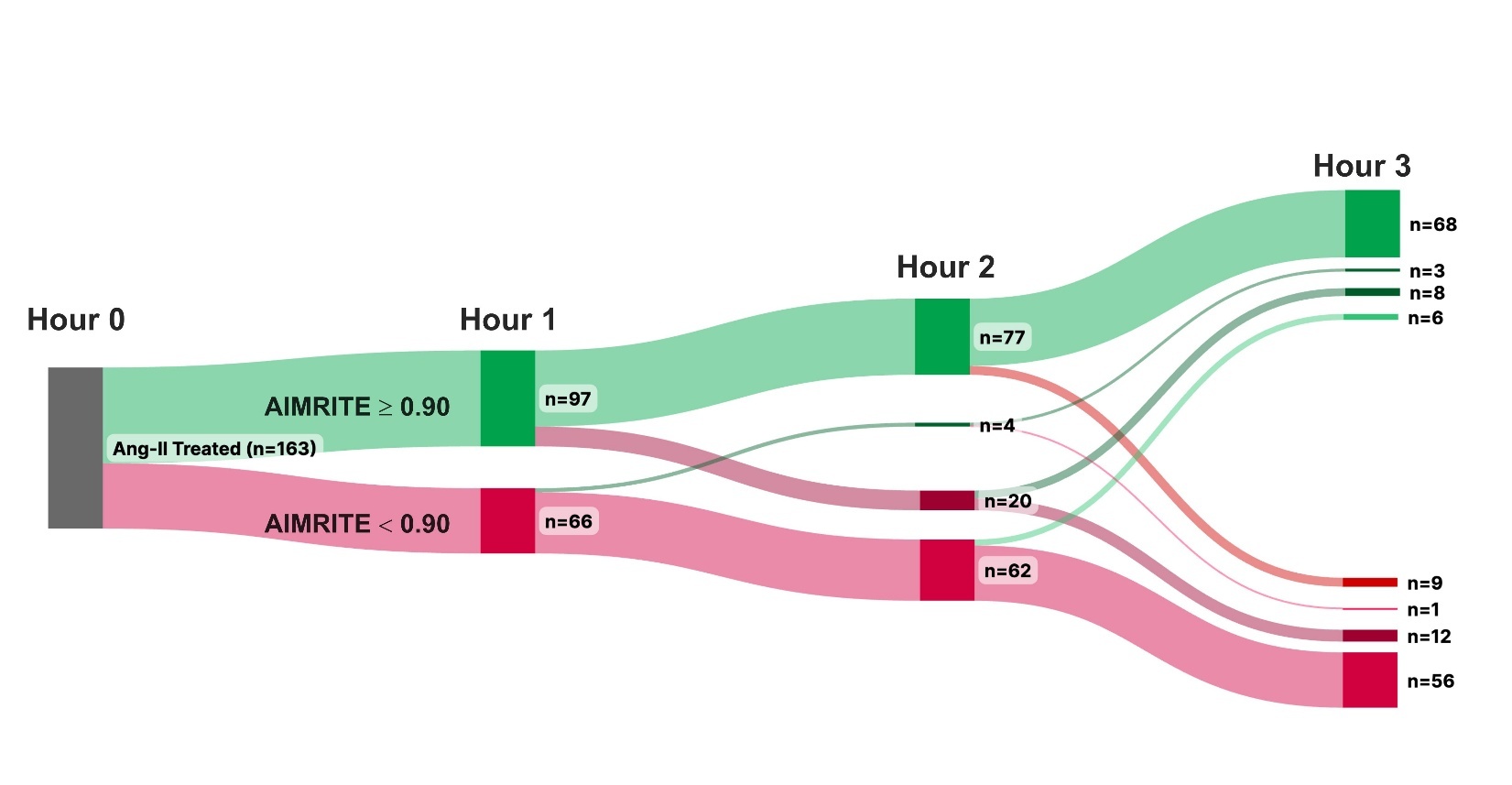
**

**Figure-s5: Alluvial Plot of AIMRITE Categorization Over the First 3 Hours**

Among the patients who received ang-II, AIMRITE categorization as responsive (green) vs. resistant (red) is shown at hours 1, 2, and 3

Abbreviations: Ang-II – Angiotensin-II; AIMRITE – Angiotensin-II Initial MAP Response Index of Treatment Effect.

**
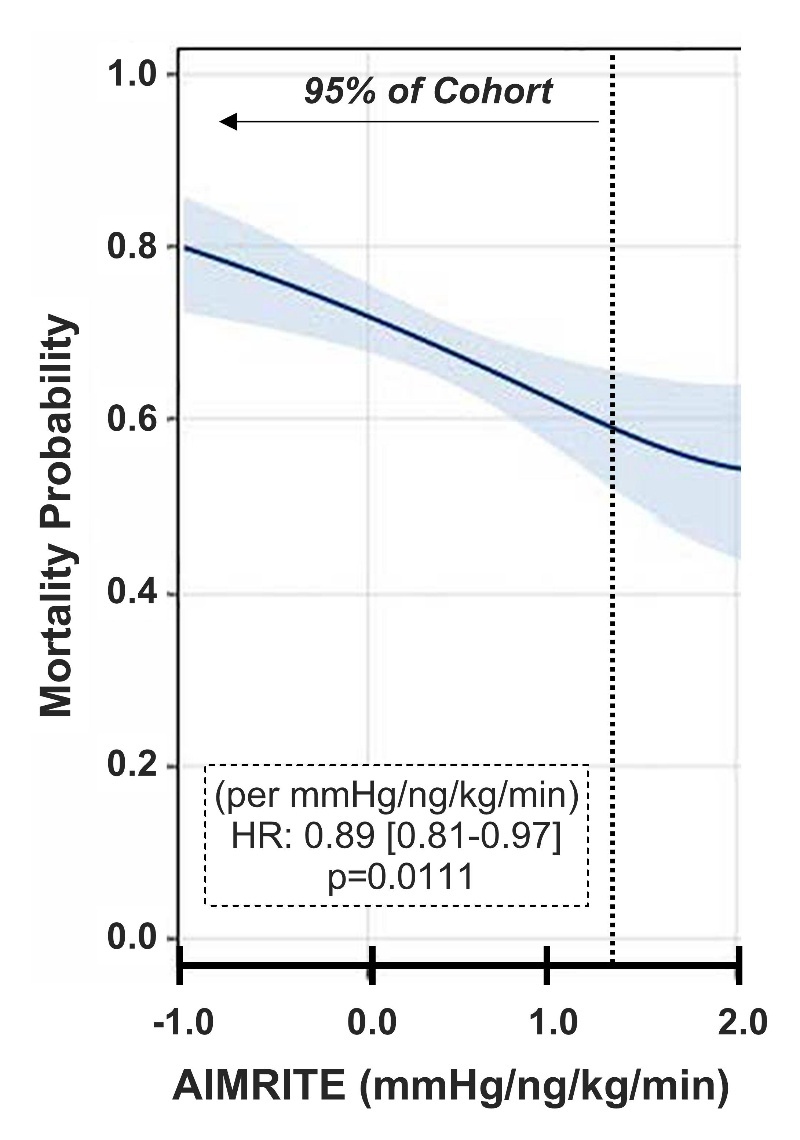
**

**Figure-s6: Association of AIMRITE with Mortality at Day-28 in an Independent Retrospective Cohort**

Day-28 mortality probability plot shows the predicted probability of death as a function of the AIMRITE (modeled non-parametrically as a continuous variable). The vertical dotted line indicates the 95^th^ percentile of AIMRITE among subjects. Shaded areas indicate 95% confidence bands. Boxed text displays the effect estimates from the Cox model with a linear predictor for comparison. Abbreviations: Ang-II – angiotensin-II, AIMRITE – angiotensin-II initial MAP response index of treatment effect, MAP – mean arterial pressure, HR – hazard ratio.

**SUPPLEMENTAL TABLES**

| **Table-s1: Explained Variance in AIMRITE from Component Variables Among Angiotensin-II Treated Patients** | | |
| --- | --- | --- |
| Component Variable | Regression Equation | r^2^ |
|  |  |  |
| X = Change in MAP at Hr_1_ | AIMRITE = -0.016 + 0.197(X) | r^2^ = 0.24 |
| X = Angiotensin-II Dose at Hr_1_ | AIMRITE = 3.072 – 0.049(X) | r^2^ = 0.24 |
| X = (Change in MAP at Hr_1_) | Ln(AIMRITE) = -1.974 + 0.872(Ln[X]) | r^2^ = 0.26 |
| X = (Angiotensin-II Dose at Hr_1_) | Ln(AIMRITE) = 2.265 – 0.483(Log_2_[X]) | r^2^ = 0.79 |
| Regression equations relating AIMRITE to its component variables, i.e., the change in MAP and the study drug dose. The regression equation shows the solution for the fitted curve of the AIMRITE as a function of the component variable (designated ‘X’). The r^2^ shows the total explained variance in the AIMRITE variable attributable to the indicated component variable, where an r^2^ = 1.00 indicates a perfect correlation and 0.00 indicates no explained variance. The observed distribution of the angiotensin-II dose suggested this relationship was better modeled on a log-scale, and the results of this transformation are shown. There was no such effect for MAP, consistent with the lack of change in r^2^.  Abbreviations: AIMRITE – Angiotensin-II Initial MAP Response Index of Treatment Effect; MAP – mean arterial pressure. | | |

| **Table-s2: Missing Data Prevalence for Key Fields** | |
| --- | --- |
| **N** | 321 |
| **Exposure Variables** |  |
| Baseline Mean Arterial Pressure* | 0 |
| Mean Arterial Pressure at hr_1_ | 0 |
| Study Drug Dose at hr_0_ | 0 |
| Study Drug Dose at hr_1_ | 0 |
| AIMRITE at hr_1_ | 0 |
| **Clinical Characteristics and Model Covariates** |  |
| Age* | 0 |
| Female* | 0 |
| Body Mass Index (kg/m^2^) | 0 |
| Cause of Vasodilatory Shock – n (%) | 0 |
| Baseline APACHE II Score* | 0 |
| Acute Respiratory Distress Syndrome at Baseline* | 0 |
| Intubated at Baseline | 0 |
| Renal Replacement Therapy at Screening | 0 |
| Baseline Norepinephrine Equivalent Dose* | 0 |
| Vasopressin use* | 0 |
| Central Venous Pressure | 72 (22.4%) |
| Cardiac Index | 179 (55.8%) |
| ScvO_2_ | 84 (26.2%) |
| Chronic Kidney Disease* | 0 |
| Chronic ACE-Inhibitor Exposure* | 0 |
| Chronic Angiotensin Receptor Blocker Exposure* | 0 |
| Albumin | 11 (3.4%) |
| Creatinine | 1 (0.3%) |
| Lactate | 36 (11.2%) |
| pH | 0 |
| **Biomarkers** |  |
| Renin Hour 0 | 43 (13.4%) |
| Renin Hour 3 | 104 (32.4%) |
| Angiotensin II Hour 0 | 37 (11.5%) |
| Angiotensin II Hour 3 | 28 (8.7%) |
| * - denotes a variable that was used as a covariate in the multivariable models assessing the association of AIMRITE with clinical outcomes. There were not any missing data for any of the outcomes assessed.  Abbreviations: AIMRITE – angiotensin II initial mean arterial pressure response index; APACHE II – Acute Physiology and Chronic Health Evaluation II Score; ACE – angiotensin Converting Enzyme. | |

| **Table -s3: Multivariable Model for Association of AIMRITE with 28-Day Mortality** | | | |
| --- | --- | --- | --- |
| **Variable** | **HR** | **95% CI** | **p-value** |
| **AIMRITE among Ang-II** _(per mmHg/ng/kg/min)_ | **0.86** | **0.76 - 0.97** | **p=0.0113** |
| AIMRITE among Placebo _(per mmHg/ng/kg/min)_ | 1.13 | 0.94 - 1.36 | p_int_=0.0145 |
| Chronic ACE-I | 0.87 | 0.46 - 1.65 | p=0.67 |
| Chronic ARB | 0.72 | 0.36 - 1.44 | p=0.35 |
| Age _(per 10 years)_ | 1.19 | 1.07 - 1.33 | p=0.0012 |
| Baseline NED _(per log-mcg/kg/min)_ | 1.55 | 1.16 - 2.09 | p=0.0035 |
| Baseline Vasopressin Use | 1.75 | 1.21 - 2.53 | p=0.0030 |
| Baseline MAP _(per mmHg)_ | 0.94 | 0.92 - 0.97 | p<0.0001 |
| Baseline APACHE-II Score _(per 1 point)_ | 1.04 | 1.02 - 1.06 | p=0.0007 |
| Female Sex | 1.12 | 0.81 - 1.56 | p=0.49 |
| ARDS at Baseline | 1.31 | 0.92 - 1.88 | p=0.14 |
| Chronic Kidney Disease | 0.69 | 0.47 - 1.01 | p=0.0531 |
| Baseline pH _(per 0.01 pH units)_ | 0.97 | 0.95 - 0.98 | p<0.0001 |
| Full multivariable model output. P_int_ refers to the p-value for an interaction term between AIMRITE and treatment assignment. For ease of interpretation, the hazard ratios and 95% CI for the AIMRITE effect are displayed at both levels of treatment.  Abbreviations: AIMRITE – Angiotensin-II Initial MAP Response Index of Treatment Effect; Ang-II – angiotensin-II; ARB – angiotensin receptor blocker; ACE-I – angiotensin-converting enzyme inhibitor; APACHE – acute physiology and chronic illness evaluation score; ARDS – acute respiratory distress syndrome; HR – hazard ratio; MAP – mean arterial pressure; NED – norepinephrine equivalent dose. | | | |

| **Table-s4: Multivariable Model for Association of AIMRITE Category with 28-Day Mortality** | | | |
| --- | --- | --- | --- |
| **Variable** | **HR** | **95% CI** | **p-value** |
| **Responsive vs. Resistant** | **0.59** | **0.36 - 0.94** | **p=0.0279** |
| **Responsive vs. Placebo** | **0.66** | **0.44 - 0.99** | **p=0.0438** |
| **Resistant vs. Placebo** | **1.12** | **0.74 - 1.69** | **p=0.58** |
| Chronic ACE-I | 0.82 | 0.43 - 1.55 | p=0.54 |
| Chronic ARB | 0.65 | 0.32 - 1.31 | p=0.23 |
| Age _(per 10 years)_ | 1.22 | 1.10 - 1.36 | p=0.0003 |
| Baseline NED _(per log-mcg/kg/min)_ | 1.58 | 1.17 - 2.12 | p=0.0025 |
| Baseline Vasopressin Use | 1.71 | 1.18 - 2.47 | p=0.0043 |
| Baseline MAP _(per mmHg)_ | 0.95 | 0.92 - 0.97 | p=0.0001 |
| Baseline APACHE-II Score _(per 1 point)_ | 1.03 | 1.01 - 1.06 | p=0.0018 |
| Female Sex | 1.06 | 0.76 - 1.48 | p=0.73 |
| ARDS at Baseline | 1.32 | 0.93 - 1.89 | p=0.13 |
| Chronic Kidney Disease | 0.67 | 0.45 - 0.98 | p=0.0397 |
| Baseline pH _(per 0.01 pH units)_ | 0.97 | 0.95 - 0.99 | p=0.0002 |
| Abbreviations: AIMRITE – Angiotensin-II Initial MAP Response Index of Treatment Effect; ARB – angiotensin receptor blocker; ACE-I – angiotensin-converting enzyme inhibitor; APACHE – acute physiology and chronic illness evaluation score; ARDS – acute respiratory distress syndrome; HR – hazard ratio; MAP – mean arterial pressure; NED – norepinephrine equivalent dose. | | | |

| **Table-s5: Multivariable Model for Association of AIMRITE with Alive and Vasopressor-Free at Day-7** | | | |
| --- | --- | --- | --- |
| **Variable** | **OR** | **95% CI** | **p-value** |
| **AIMRITE among Ang-II** _(per mmHg/ng/kg/min)_ | **1.45** | **1.18 - 1.77** | **p=0.0003** |
| AIMRITE among Placebo _(per mmHg/ng/kg/min)_ | 0.73 | 0.37 - 1.47 | p_int_=0.0648 |
| Chronic ACE-I | 1.28 | 0.49 - 3.37 | p=0.62 |
| Chronic ARB | 2.50 | 0.86 - 7.27 | p=0.0918 |
| Age _(per 10 years)_ | 0.93 | 0.78 - 1.10 | p=0.38 |
| Baseline NED _(per log-mcg/kg/min)_ | 0.63 | 0.40 - 1.00 | p=0.0478 |
| Baseline Vasopressin Use | 0.47 | 0.27 - 0.82 | p=0.0076 |
| Baseline MAP _(per mmHg)_ | 1.12 | 1.06 - 1.19 | p=0.0002 |
| Baseline APACHE-II Score _(per 1 point)_ | 0.97 | 0.93 - 1.00 | p=0.0463 |
| Female Sex | 0.69 | 0.41 - 1.17 | p=0.16 |
| ARDS at Baseline | 1.00 | 1.00 - 1.00 | p=0.43 |
| Chronic Kidney Disease | 1.90 | 1.02 - 3.54 | p=0.0448 |
| Baseline pH _(per 0.01 pH units)_ | 1.04 | 1.01 - 1.07 | p=0.0178 |
| Full multivariable model output. P_int_ refers to the p-value for an interaction term between AIMRITE and treatment assignment. For ease of interpretation, the hazard ratios and 95% CI for the AIMRITE effect are displayed at both levels of treatment.  Abbreviations: AIMRITE – Angiotensin-II Initial MAP Response Index of Treatment Effect; ARB – angiotensin receptor blocker; ACE-I – angiotensin-converting enzyme inhibitor; APACHE – acute physiology and chronic illness evaluation score; ARDS – acute respiratory distress syndrome; MAP – mean arterial pressure; NED – norepinephrine equivalent dose; OR – odds ratio. | | | |

| **Table-s6: Multivariable Model for Association of AIMRITE Category with Alive and Vasopressor-Free at Day-7** | | | |
| --- | --- | --- | --- |
| **Variable** | **OR** | **95%CI** | **p-value** |
| **Responsive vs. Resistant** | **3.08** | **1.44 - 6.58** | **p=0.0038** |
| **Responsive vs. Placebo** | **1.71** | **0.94 - 3.11** | **p=0.0782** |
| **Resistant vs. Placebo** | **0.56** | **0.28 - 1.10** | **p=0.0933** |
| Chronic ACE-I | 1.31 | 0.51 - 3.37 | p=0.57 |
| Chronic ARB | 2.76 | 0.95 - 8.02 | p=0.0627 |
| Age _(per 10 years)_ | 0.89 | 0.75 - 1.07 | p=0.21 |
| Baseline NED _(per log-mcg/kg/min)_ | 0.62 | 0.39 - 0.97 | p=0.0356 |
| Baseline Vasopressin Use | 0.51 | 0.3.0 - 0.88 | p=0.0149 |
| Baseline MAP _(per mmHg)_ | 1.11 | 1.05 - 1.18 | p=0.0004 |
| Baseline APACHE-II Score _(per 1 point)_ | 0.97 | 0.93 – 0.99 | p=0.0327 |
| Female Sex | 0.75 | 0.44 - 1.26 | p=0.27 |
| ARDS at Baseline | 0.68 | 0.37 - 1.25 | p=0.21 |
| Chronic Kidney Disease | 1.85 | 1.00 - 3.42 | p=0.0504 |
| Baseline pH _(per 0.01 pH units)_ | 1.03 | 1.00 - 1.06 | p=0.0440 |
| Abbreviations: AIMRITE – Angiotensin-II Initial MAP Response Index of Treatment Effect; ARB – angiotensin receptor blocker; ACE-I – angiotensin-converting enzyme inhibitor; APACHE – acute physiology and chronic illness evaluation score; ARDS – acute respiratory distress syndrome; MAP – mean arterial pressure; NED – norepinephrine equivalent dose; OR – odds ratio. | | | |

| **Table-s7: Sensitivity Analyses for Association of AIMRITE with Mortality at Day-28** | | | |
| --- | --- | --- | --- |
| **Subset** | **Hazard Ratio** | **95% CI** | **p-value** |
| **Primary Analysis** |  |  |  |
| AIMRITE among Ang-II | 0.86 | 0.76 - 0.97 | p=0.0113 |
| AIMRITE among Placebo | 1.13 | 0.94 - 1.36 | p_int_=0.0145 |
| **Excluding ARB or ACEi Exposed** | |  |  |
| AIMRITE among Ang-II | 0.87 | 0.77 - 0.99 | p=0.0300 |
| AIMRITE among Placebo | 1.13 | 0.93 - 1.37 | p_int_=0.0246 |
| **Excluding CPB-Associated Vasoplegia** | |  |  |
| AIMRITE among Ang-II | 0.86 | 0.77 - 0.97 | p=0.0133 |
| AIMRITE among Placebo | 1.12 | 0.93 - 1.34 | p_int_=0.0203 |
| **Including Definite and Possible Septic Shock Only** | |  |  |
| AIMRITE among Ang-II | 0.87 | 0.77 - 0.97 | p=0.0176 |
| AIMRITE among Placebo | 1.11 | 0.92 - 1.35 | p_int_=0.0295 |
| **Including Definite Septic Shock Only** |  |  |  |
| AIMRITE among Ang-II | 0.87 | 0.77 - 0.97 | p=0.0169 |
| AIMRITE among Placebo | 1.11 | 0.92 - 1.35 | p_int_=0.0302 |
| **Excluding Baseline NED ≥ 0.5 mcg/kg/min** | |  |  |
| AIMRITE among Ang-II | 0.87 | 0.76 - 0.99 | p=0.0403 |
| AIMRITE among Placebo | 1.12 | 0.93 - 1.35 | p_int_=0.0290 |
| **Excluding ARB or ACEi Exposed, CPB-Associated Vasoplegia, and Baseline NED ≥ 0.5 mcg/kg/min** | | | |
| AIMRITE among Ang-II | 0.87 | 0.76 - 1.01 | p=0.0695 |
| AIMRITE among Placebo | 1.09 | 0.89 - 1.32 | p_int_=0.0769 |
| Effect sizes reported from multivariable models that adjusted for age, sex, chronic angiotensin receptor blocker use, chronic angiotensin-converting enzyme inhibitor use, and chronic kidney disease, as well as baseline APACHE-II score, MAP, total vasopressor dose in norepinephrine equivalent-dose (NED), use of vasopressin, arterial pH, acute respiratory distress syndrome, and use of renal replacement therapy.  Abbreviations: AIMRITE – Angiotensin-II Initial MAP Response Index of Treatment Effect; Ang-II – Angiotensin-II; ARB – Angiotensin Receptor Blocker; ACEi – Angiotensin-Converting Enzyme Inhibitor; CPB – Cardiopulmonary Bypass; NED – Norepinephrine Equivalent Dose | | | |

| **Table-s8: Sensitivity Analyses for Association of AIMRITE Response Categories with Mortality at Day-28** | | | |
| --- | --- | --- | --- |
| **Subset** | **Hazard Ratio** | **95% CI** | **p-value** |
| **Primary Analysis** |  |  |  |
| Responsive vs. Resistant | 0.59 | 0.36 - 0.94 | p=0.0279 |
| Responsive vs. Placebo | 0.66 | 0.44 - 0.99 | p=0.0438 |
| Resistant vs. Placebo | 1.12 | 0.74 - 1.69 | p=0.58 |
| **Excluding ARB or ACEi Exposed** |  |  |  |
| Responsive vs. Resistant | 0.65 | 0.39 - 1.07 | p=0.0921 |
| Responsive vs. Placebo | 0.66 | 0.43 – 1.00 | p=0.0511 |
| Resistant vs. Placebo | 1.02 | 0.64 - 1.61 | p=0.93 |
| **Excluding CPB-Associated Vasoplegia** |  |  |  |
| Responsive vs. Resistant | 0.58 | 0.36 - 0.95 | p=0.0287 |
| Responsive vs. Placebo | 0.67 | 0.44 - 1.01 | p=0.0582 |
| Resistant vs. Placebo | 1.15 | 0.76 - 1.74 | p=0.51 |
| **Including Definite and Possible Septic Shock Only** | |  |  |
| Responsive vs. Resistant | 0.58 | 0.34 - 0.97 | p=0.0362 |
| Responsive vs. Placebo | 0.72 | 0.46 - 1.12 | p=0.14 |
| Resistant vs. Placebo | 1.25 | 0.80 - 1.95 | p=0.33 |
| **Including Definite Septic Shock Only** |  |  |  |
| Responsive vs. Resistant | 0.58 | 0.34 - 0.97 | p=0.0362 |
| Responsive vs. Placebo | 0.72 | 0.46 - 1.12 | p=0.14 |
| Resistant vs. Placebo | 1.25 | 0.80 - 1.95 | p=0.33 |
| **Excluding Baseline NED ≥ 0.5 mcg/kg/min** | |  |  |
| Responsive vs. Resistant | 0.60 | 0.33 - 1.11 | p=0.1029 |
| Responsive vs. Placebo | 0.53 | 0.33 - 0.86 | p=0.0104 |
| Resistant vs. Placebo | 0.88 | 0.50 - 1.55 | p=0.65 |
| **Excluding ARB or ACEi Exposed, CPB-Associated Vasoplegia, and Baseline NED ≥ 0.5 mcg/kg/min** | | | |
| Responsive vs. Resistant | 0.65 | 0.34 - 1.27 | p=0.21 |
| Responsive vs. Placebo | 0.60 | 0.36 - 0.98 | p=0.0432 |
| Resistant vs. Placebo | 0.91 | 0.48 - 1.73 | p=0.78 |
| Effect sizes reported from multivariable models that adjusted for age, sex, chronic angiotensin receptor blocker use, chronic angiotensin-converting enzyme inhibitor use, and chronic kidney disease, as well as baseline APACHE-II score, MAP, total vasopressor dose in norepinephrine equivalent-dose (NED), use of vasopressin, arterial pH, acute respiratory distress syndrome, and use of renal replacement therapy.  Abbreviations: AIMRITE – Angiotensin-II Initial MAP Response Index of Treatment Effect; Ang-II – Angiotensin-II; ARB – Angiotensin Receptor Blocker; ACEi – Angiotensin-Converting Enzyme Inhibitor; CPB – Cardiopulmonary Bypass; NED – Norepinephrine Equivalent Dose | | | |

| **Table-s9: Serial Calculation of AIMRITE Over First 3 Hours and Association with 28-Day Mortality** | | | | | | |
| --- | --- | --- | --- | --- | --- | --- |
|  | Unadjusted | | | Multivariable | | |
| **Subset** | **HR** | **95% CI** | **p-value** | **HR** | **95% CI** | **p-value** |
| *Continuous AIMRITE Variable* |  |  |  |  |  |  |
| Hour 1 _(per mmHg/ng/kg/min)_ | 0.84 | 0.74 - 0.95 | p=0.0044 | 0.86 | 0.76 - 0.97 | p=0.0113 |
| Hour 2 _(per mmHg/ng/kg/min)_ | 0.83 | 0.72 - 0.94 | p=0.0044 | 0.83 | 0.75 - 0.95 | p=0.0051 |
| Hour 3 _(per mmHg/ng/kg/min)_ | 0.84 | 0.74 - 0.95 | p=0.0043 | 0.84 | 0.78 - 0.97 | p=0.0132 |
| *Categorized AIMRITE Variable* |  |  |  |  |  |  |
| Hour 1 _Responsive vs. Resistant_ | 0.49 | 0.31 - 0.78 | p=0.0023 | 0.58 | 0.35 - 0.96 | p=0.0334 |
| Hour 2 _Responsive vs. Resistant_ | 0.44 | 0.28 - 0.71 | p=0.0007 | 0.56 | 0.34 - 0.93 | p=0.0246 |
| Hour 3 _Responsive vs. Resistant_ | 0.47 | 0.29 - 0.74 | p=0.0012 | 0.58 | 0.35 - 0.96 | p=0.0336 |
| Multivariable models adjusted for age, sex, chronic angiotensin receptor blocker use, chronic angiotensin-converting enzyme inhibitor use, and chronic kidney disease, as well as baseline APACHE-II score, MAP, total vasopressor dose in norepinephrine equivalent-dose (NED), use of vasopressin, arterial pH, acute respiratory distress syndrome, and use of renal replacement therapy.  Abbreviations: AIMRITE – Angiotensin-II Initial MAP Response Index of Treatment Effect; Ang-II – angiotensin-II; ARB – angiotensin receptor blocker; ACE-I – angiotensin-converting enzyme inhibitor; APACHE – acute physiology and chronic illness evaluation score; ARDS – acute respiratory distress syndrome; HR – hazard ratio; MAP – mean arterial pressure; NED – norepinephrine equivalent dose. | | | | | | |
